# Effects of fecal microbiota transplantation from yaks on weaning diarrhea, fecal microbiota composition, microbial network structure and functional pathways in Chinese Holstein calves

**DOI:** 10.3389/fmicb.2022.898505

**Published:** 2022-09-23

**Authors:** Yuanyuan Li, Xin Li, Yanyan Wu, Wenju Zhang

**Affiliations:** ^1^College of Animal Science and Technology, Shihezi University, Shihezi, China; ^2^College of Life Sciences, Shihezi University, Shihezi, China

**Keywords:** fecal microbiota transplantation, post-weaning diarrhea, fecal microbiota, microbial network structure, functional prediction, calf

## Abstract

This study was conducted to investigate the effect of fecal microbiota transplantation (FMT) from yaks on weaning diarrhea, fecal microbiota composition, microbial network structure and functional pathways in Chinese Holstein Calves. In this study, 50 calves were randomly divided into five groups of 10 each: NC group (no supplementation), Control group (normal saline), low concentration FMT group (LFMT, 1 × 10^8^ CFU/ml), high concentration FMT group (HMFT, 1 × 10^9^ CFU/ml), and sterilized FMT group (SMFT, sterilized bacterial solution). The test lasted for 30 days. We found that FMT reduced the incidence of diarrhea in weaned calves, and the anti-diarrhea effect of LFMT was stronger than those of HFMT and SFMT. Calf feces were collected by rectal palpation on days 5, 10, 15, and 20 post-weaning, and high-throughput sequencing of bacterial 16S rRNA and fungal internal transcribed spacer region of fecal microbiota was performed. We observed that the richness and diversity of bacterial microbiota in the LFMT, HFMT, and SFMT groups were higher than those in the NC and Control groups at day 20 after weaning. The treatment had a significant effect on bacterial richness (*p* < 0.05), but not on fungal diversity or richness. The analysis of gut microbiome showed that *Firmicutes* and *Bacteroides* were the main bacterial phyla in the feces of weaned calves, and *norank*_ *f Muribaculaceae*, *UCG-005*, *Rikenellaceae*_*RC9_gut_group*, *Bacteroides*, and *Blautia* were the main genera. *Ascomycota* and *Basidiomycota* were the main fungal phyla. Compared to abundance parameters in the Control and NC groups, relative abundances of *Firmicutes* in the FMT groups increased at different time points after weaning. The relative abundance of *Blautia* and *Lactobacillus* in the LFMT group increased significantly after weaning. In addition, abundances of *Ruminococcus* and *Romboutsia*, which produce short-chain fatty acids, were also increased in different FMT groups. FMT significantly increased the relative abundance of beneficial bacteria, enhanced the complexity of the fecal microbial network, and promoted important metabolic and cellular processes in weaned calves. In conclusion, our study provides a reference and theoretical basis for FMT to prevent calf weaning diarrhea and other intestinal diseases in ruminants.

## Introduction

Weaning is one of the most stressful periods in calf’s life (Clark et al., 2016). Owing to the incomplete development of gastrointestinal system and intestinal microbial ecosystem of weaned calves, weaning stress occurs in the face of sudden changes in feeding environment (single column to group feeding) and diet, leading to gastrointestinal dysfunction and diarrhea (Wang et al., 2021). The high incidence of diarrhea in weaned calves is a major cause of stunted growth and mortality, which severely affects calf welfare and causes substantial economic losses to cattle industry (Pempek et al., 2019). Therefore, post-weaning diarrhea of calves has become an urgent problem that needs to be solved to promote the development of bovine husbandry.

Currently, antibiotics are widely used in animal husbandry to treat or prevent diarrhea and promote livestock growth (Brown et al., 2017). However, many studies have shown that the long-term use of antibiotics not only disrupts the normal community structure of animal gut microbes, but also increases the presence of disease and drug-resistant bacteria in the gut (Listed, 2014).In addition, cattle are food-producing animals, so residual antibiotics in the body may also seriously affect food safety and pose a great threat to human health (Zhang et al., 2019; Shao et al., 2021). Therefore, finding green and pollution-free antibiotics alternatives to prevent diarrhea in early weaned calves is crucial for animal husbandry development and food safety.

The animal gut contains many complex microorganisms, including bacteria, fungi, viruses, which are closely related to host health. It has been established that rebuilding a healthy gut microbial community is an effective way to prevent or treat gastrointestinal diseases in animals (McKenney and Pamer, 2015). During fecal microbiota transplantation (FMT), the functional microbiota in the fecal bacterial fluid of healthy donors is transplanted into the gastrointestinal tract of a patient to treat diseases by restoring the normal intestinal microbiota structure (He et al., 2017). This approach has been clinically used in humans as a highly effective treatment for inflammatory bowel disease, irritable bowel syndrome, metabolic syndrome, and other conditions (Smits et al., 2013; Leshem et al., 2019). More and more studies have shown that FMT also has good application potential in livestock and poultry production. For example, FMT effectively improved the intestinal microbiota structure and intestinal epithelial barrier function of calves with passive immunity failure (Wu, 2018). Furthermore, altering the gut microbiota of pre-weaned calves treated diarrhea and positively affected calf performance and health (Kim et al., 2021).

However, to the best of our knowledge, the application of FMT for the prevention of post-weaning diarrhea in calves has not been reported. In addition, the exact mechanism of FMT positive effects is unclear. Therefore, in this study, we sought to establish if transplantation of fecal microbiota from healthy donor yaks can prevent post-weaning diarrhea in recipient Chinese Holstein calves. Yaks are herbivorous ruminants, mainly living in high mountains with an average altitude of more than 3,000 m centered on the Qinghai Tibet Plateau of China. Yaks have strong adaptability and disease resistance. Our previous studies showed that the changes in the composition and structure of fecal micromicrobiota before and after weaning of yaks were helpful for yaks to adapt to high altitude, high cold and free grazing, and reduce the occurrence of weaning stress. At present, there are few studies on bovine FMT, and there are few studies on cross species transplantation of different kinds of bovine intestinal microorganisms to analyze their functions. We speculate that if the functional microbiota in yak feces can be transplanted into the intestines of Holstein calves, it may help to improve the intestinal microbiota and reduce calf diarrhea caused by weaning stress. Therefore, this study takes yaks as FMT donors, and analyzes the impact of FMT on the structure of calf fecal bacteria through 16S rRNA amplicon gene sequencing. We analyzed the effect of FMT on the structure of calf fecal bacterial microbiota by 16S rRNA amplicon gene sequencing. In addition, given that an increasing number of studies show that fungi play an important role in animal intestinal health (Fiers et al., 2020; Doron et al., 2021), we also studied the effect of FMT on the structure of fecal fungal microbiota by sequencing the amplified fungal internal transcribed spacer (ITS) region in the sample group with low diarrhea rate and in control animals. Finally, the effect of FMT on the structure and potential function of calf intestinal microbial network was analyzed, which provided new insights for understanding possible ways to prevent diarrhea in weaned calves and establish a theoretical basis for the application of FMT in ruminants.

## Materials and methods

### Ethics statement

Animal experiments were conducted in compliance with the regulations of the Administration of Affairs Concerning Experimental Animals (Ministry of Science and Technology, China; revised in June 2004). This study was approved by the Bioethics Committee of the Shihezi University (no. A2021-32).

### Preparation of bacterial suspension for fecal microbial transplantation

FMT donor screening in this study was carried out using the criteria of human clinical FMT and porcine FMT donor screening (Cammarota et al., 2017; Hu et al., 2018a). Weaned yaks in Xinjiang, China, which had not been treated with antibiotics within 1 month and had no abnormalities in genetic background, drug history, medical history, growth development, behavioral characteristics, or presence of common pathogens and infectious diseases, were selected as fecal donors. The structure of fecal microbiota in yaks is shown in Supplementary Table 1. The preparation of bacterial suspension of donor yak feces (Hamilton et al., 2012), the fecal homogenization solution was filtered through sterile medical gauze and a stainless-steel sieve to remove large particles, after which it was further filtered through 2.0, 1.0, 0.5, and 0.25-mm stainless steel sieves to remove food residues and small particles in the feces. The filtrate was then collected. The obtained filtrate was transferred into a sterile centrifuge tube and centrifuged at 4°C, 6,000 × *g* for 15 min. The supernatant was decanted, and 2 × volume normal saline was added to the precipitate and centrifuged again. The process was repeated 3 times to make a uniform, colorless, and odorless fecal suspension. Viable bacteria of the filtered fecal bacterial liquid were counted in the Petroff Hauser chamber using optical microscope and methylene blue staining. The concentration of bacteria in the suspension was adjusted to 1 × 10^8^ CFU/ml or 1 × 10^9^ CFU/ml, according to the counting results, and finally, sterile glycerol was added to a concentration of 10%, and 5 ml samples were frozen at −80°C in a cryovial until use.

### Feeding of FMT recipient cattle

Fifty Chinese Holstein calves 50 ± 3 days in age and similar body weight (78.85 ± 2.56 kg) were selected and randomly divided into five groups of 10 calves each. Recipient cattle were screened according to the Supplementary Table 2 criteria. To avoid cross-contamination, all calves were housed in calf islands (1.8 × 1.4 × 1.2 m). Then, at 07:00 and 18:00 every day, the calves were fed twice from two equal volume plastic buckets. From day 50 to day 56, the amount of milk was 5 l/days (2.5 l/meal). On days 57–60, the calves were fed once a day, and the milk volume was reduced to 1 l/day. After weaning on day 61, the calves were not fed milk. Feeding milk was produced by the same farm and pasteurized at 60°C for 1 h before use. The starter concentrate was provided by Xinjiang Urumqi Chia Tai Feed Co., Ltd. (Urumqi, China), and the calves were fed from postnatal day 4. All calves had free access to water and starter concentrate. The composition of the starter concentrate is shown in Supplementary Table 3.

### FMT experiment design and sample collection

From postnatal day 50 to postnatal day 60, group 1 was fed normally, without any treatment, so it was the negative control (NC) group. Group 2 was given 5 ml of sterile normal saline every other day during morning feeding (Control group). Group 3 was given 5 ml of a 1 × 10^8^ CFU/ml fecal bacterial suspension every other day during morning feeding. This group was marked as low concentration FMT (LFMT) group. Group 4 was given 5 ml of a 1 × 10^9^ CFU/ml fecal bacterial suspension every other day during morning feeding and marked as high concentration FMT (HFMT) group. Group 5 was given 5 ml of bacterial liquid sterilized at 103 kPa and 121°C for 15 min on the next day. This group was marked as sterilized FMT (SFMT) group. All fecal bacterial fluid samples were revived in a 37°C water bath before administration. In order to prevent the inactivation of fecal microorganisms affected by gastric acid, sterile sodium bicarbonate solution was added to the fecal bacterial suspension after resuscitation before feeding, and the final concentration of sodium bicarbonate was 1.2%. In addition, fecal bacterial suspension was fed in a bottle to achieve the purpose of fecal bacterial transplantation and intestinal microbiota regulation (Hamilton et al., 2012; Wu, 2018). The experiment lasted for 30 days. The entire study was conducted at the Tianjin Farm of Tianshan Military Reclamation Company in Shihezi, Xinjiang, China from April to June 2021.

Before morning feeding on the 5th, 10th, 15th, and 20th day after weaning, five calves were selected from the NC, Control, LFMT, HFMT and SFMT groups respectively, and their feces were collected by rectal palpation. The fresh feces were placed into 2 ml frozen tubes sterilized by high temperature and high pressure. Three frozen tubes were collected from each calf, and fecal sample of each frozen tube was about 0.5 g. The frozen tube containing fecal samples was quickly frozen in liquid nitrogen and stored at −80°C after being transported to the laboratory for analysis.

### Analysis of the diarrhea rate and diarrhea index

From the day of weaning to the 20th day after weaning, the feces of each calf in each group were scored with reference to the diarrhea index scoring method at 09:00 every morning as follows: normal feces (solid) were scored as 0 points; wet feces (semi-solid)—as 1 point; mild diarrhea (mushy stool)—as 2 points; severe diarrhea (watery stool)—as 3 points. Scores indicating mild or severe diarrhea were considered to signify diarrhea occurrence. The numbers of days and calves with diarrhea during the test period were recorded for diarrhea index and diarrhea rate statistics (Mcguirk, 2008). The following formulas were used to calculate the diarrhea rate and diarrhea index for each group of calves:

Diarrhea rate (%) = ∑number of calves with diarrhea in each group × number of days with diarrhea/(test days × number of calves in each group) × 100%.

Diarrhea index = Sum of diarrhea scores of calves in each group during the test period/(test days × number of calves in each group).

### High-throughput sequencing of bacterial 16S rRNA gene and fungal its region

Total microbial community genomic DNA was extracted from stool samples according to the instructions for the use of the E.Z.N.A.^®^ soil DNA Kit (Omega Bio-Tek, Norcross, GA, United States). DNA purity and concentration were determined using a NanoDrop2000 ultra-micro spectrophotometer (Thermo Fisher Scientific, Wilmington, United States); DNA integrity was detected by 1% agarose gel electrophoresis at a voltage of 5 V/cm for 20 min.

Bacterial primers 338F (ACTCCTACGGGAGGCAGCAG) and 806R (GGACTACHVGGGTWTCTAAT) were used to amplify the V3–V4 region of the bacterial 16S rRNA gene (Guo et al., 2018). The primer sequences for the amplification of the fungal ITS1F–ITS2R region were F: 5′-CTTGGTCATTTAGAGGAAGTAA-3′ and R: 5′-GCTGCGTTCTTCATCGATGC-3′ (Zhu et al., 2019). Three PCR replicates were performed for each sample. The PCR products of the three replicates were mixed, and the product was extracted from 2% agarose gel and purified using the AxyPrep DNA Gel Extraction Kit (Axygen Biosciences, Union City, CA, United States), then detected by 2% agarose gel electrophoresis and quantified with Quantus™ Fluorometer (Promega, United States). The Illumina’s Miseq PE300 platform (Illumina, San Diego, United States) was used for sequencing at Shanghai Meiji Biomedical Technology Co., Ltd. (Shanghai, China).

### Bioinformatics analysis

To obtain high quality and accurate results for bioinformatics analysis, quality control and filtering of the original data were carried out. The quality of the original sequence was controlled by fastp software (Chen et al., 2018; version 0.20.0).^1^ Flash software (Mago and Salzberg, 2011; version 1.2.7)^2^ was used to splice the sequences. Using uparse software (Edgar, 2013; version 7.1),^3^ we performed operational taxonomic unit (OTU) clustering of bacterial and fungal sequences according to a similarity threshold of 97% (Stackebrandt and Goebel, 1994). We also used Silva 16S rRNA database (v138) and unite ITS fungal database (release 8.0),^4^ removed singletons^5^ and applied the Bayesian algorithm with the Ribosomal Database Project classifier (version 2.2)^6^ to perform taxonomic analysis of OTU representative sequences at a similarity threshold of 97%. Then, the sparse curve was drawn based on the Chao and Shannon indices by using R language tool through the Meiji bioscience cloud platform. Using the Bray–Curtis distance algorithm and the Vegan package in R software for the analysis of similarities (ANOSIM), we assessed the similarity of community composition between all groups. Based on the partial least squares regression model, the partial least squares discriminant analysis (PLS-DA) was performed to determine the community structure data. According to the results of taxonomic analysis, the R language tool (version 3.3.1) was used to draw the column diagram of the species composition of fecal microorganisms at the phylum and genus levels. Colony composition and abundance of each group of microorganisms at the phylum and genus levels were presented by bar diagrams, and the phyla and genera with significant differences in fungi were determined by the Kruskal–Wallis *H*-test. According to the one-against-all comparison strategy, taking LDA score > 3 (*p* < 0.05) as the critical value, the species with significant differences in abundance between groups were found using the linear discriminant analysis Effect Size (LEfSe). The correlation network of fecal microorganisms at the genus level was constructed by NetworkX software to analyze the impact of FMT on species interaction. The functions of the bacterial 16S rRNA gene sequence and fungal ITS gene sequence were predicted based on PICRUSt2 (phylogenetic investigation of communities by reconstruction of unobserved states) software.

### Statistical analysis

Sequence data analysis was mainly carried out by Miseq PE300 and R software package. The diarrhea rate and diarrhea index were analyzed by one-way analysis of variance using SPSS 26.0 software. Fungal Alpha diversity index was analyzed using the independent samples *t*-test. Data are expressed as the mean ± standard error of the mean. Effects were considered statistically significant if *p* < 0.05. The fecal microbial data of calves over the whole experimental period were used as repeated measures data for analysis using the Proc Mixed model in SAS9.2. Fixed effects included experimental treatment, time, and experimental treatment × time interaction. Individual calf data in each treatment were used for the analysis.

## Results

### FMT effect on the diarrhea rate

Fecal morphology was monitored from day 1 to day 20 after weaning in calves of all five experimental groups. The diarrhea rate and diarrhea index of calves on days 1–5, 6–10, 11–15, and 16–20 after weaning are shown in Table 1. We found that the diarrhea index and diarrhea rate of calves in the FMT groups were lower than those in the NC and Control groups 10 days after weaning (*P <* 0.01). The diarrhea rate and diarrhea index of the HFMT group and LFMT group were lower than that of other groups at days 11–15 after weaning (NC vs. LFMT, *p* = 0.046). During the whole experiment, the total diarrhea rate of the LFMT group was low. The above results showed that FMT alleviated the symptoms of weaning diarrhea in calves, and the effects of the two concentrations of fecal microbiota were different.

**Table 1:** Effects of different FMT treatments on diarrhea incidence and diarrhea index of weaned calves at different time points after weaning.

Item	Experimental treatments					SEM^1^	*p*
	NC	Control	LFMT	HFMT	SFMT		
Incidence of diarrhea (1–5 days), %	24.44	20.00	12.00	14.00	15.00	2.13	0.361
Incidence of diarrhea (6–10 days), %	13.33^a,b^	24.44^a^	4.00^b^	16.00^a,b^	5.00^b^	2.64	0.069
Incidence of diarrhea (11–15 days), %	11.11	11.11	8.00	8.00	12.50	2.01	0.846
Incidence of diarrhea (16–20 days), %	17.78^a,b^	6.67^a,b^	2.00^b^	4.00^a,b^	12.50^a^	2.10	0.09
Diarrhea index (1–5 days)	0.80	0.78	0.52	0.48	0.53	0.07	0.393
Diarrhea index (6–10 days)	0.67^a^	0.98^a^	0.18^c^	0.66^a,b^	0.25^b,c^	0.08	0.002
Diarrhea index (11–15 days)	0.58	0.47	0.38	0.42	0.53	0.04	0.568
Diarrhea index (16–20 days)	1.02^a^	0.64^a,b^	0.12^c^	0.12^c^	0.50^b^	0.08	< 0.01

Experimental treatments: NC, no supplementation; Control, normal saline; LFMT, 1 × 10^8^ CFU/ml bacterial solution/calf/time; HFMT, 1 × 10^9^ CFU/ml bacterial solution/calf/time; SFMT, sterilized bacterial solution. ^a,b,c^In the same row, values with different letter superscripts mean significant difference (*p* < 0.05).

1 SEM, standard error of mean.

### Sequencing results

In this study, we investigated the effect of FMT on fecal microbiota communities in calves at four post-weaning time points (days 5, 10, 15, and 20 post-weaning) through 16S rRNA and ITS region amplification and sequence analysis. A total of 4,716,547 high-quality sequences of 16S rRNA genes were obtained from post-weaning calf fecal samples. The average number of high-quality sequences per sample was 47,165 (32,101–64,932), and the average sequence length was 415 bp. In addition, 2,831,345 optimized sequences of the ITS region were obtained, with an average number of 80,895 (32,597–114,298) sequences per sample and an average sequence length of 232 bp. All sequences were clustered into 1,944 bacterial and 708 fungal OTUs based on the 97% sequence similarity threshold.

**Figure 1:**
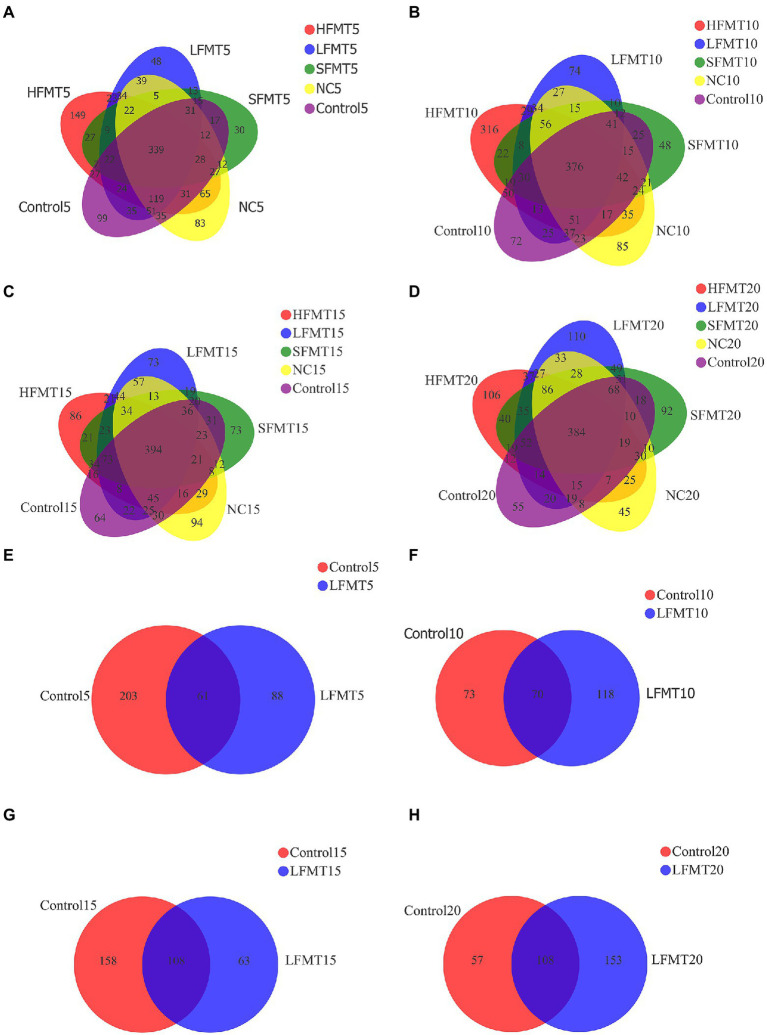
Venn diagrams were used to show the number of OTUs common or unique by bacteria and fungi at four time points after weaning of Holstein calves treated with different FMT. **(A)** Venn diagram of fecal bacteria on the 5th day after weaning. **(B)** Venn diagram of fecal bacteria on the 10th day after weaning. **(C)** Venn diagram of fecal bacteria on the 15th day after weaning. **(D)** Venn diagram of fecal bacteria on the 20th day after weaning. **(E)** Venn diagram of fecal fungi on the 5th day after weaning. **(F)** Venn diagram of fecal fungi on the 10th day after weaning. **(G)** Venn diagram of fecal fungi on the 15 h day after weaning. **(H)** Venn diagram of fecal fungi on the 20th day after weaning.

The total number of bacterial core OTUs in NC, Control, LFMT, HFMT and SFMT groups at 5, 10, 15 and 20 days after weaning were 339, 376, 394 and 384, respectively. In addition, at different time points after weaning, 83, 85, 94 and 45 OTUs unique to group NC respectively, 99, 72, 84 and 55 OTUs unique to group Control respectively, 48, 74, 73 and 110 OTUs unique to group LFMT respectively, 149, 316, 86 and 106 OTUs unique to group HFMT, respectively, and 30, 48, 73 and 92 OTUs unique to group SFMT, respectively. In fungal analysis, there were 61, 70, 108 and 108 core OTUs in all samples at 4 time points after weaning. The specific OTUs of Control group were 203, 73, 158 and 57, and that of LFMT group were 88, 118, 63 and 153 (Figure 1). The NC containing the unique OTUs mostly annotated to *Rikenellaceae_RC9_gut_group*, etc. The Control containing the unique OTUs mostly annotated to *Prevotella*, etc. The LFMT contains the unique OTUs mostly annotated to *Lactobacillus*, etc. The main annotation of HFMT specific OTUs are *Lysobacter_ deserti*, *uncultured_ bacterium_ g__ Fermentimonas* and *uncultured_ bacterium_ g__ Eubacterium_ ruminantium_Group*, etc. The main annotation of OTUs specific to SFMT are *Clostridiaceae_bacterium_DJF_VR76*, *Ruminococcus_sp._HUN007* and an*uncultured_bacterium_ g__Prevotella*.

**Figure 2:**
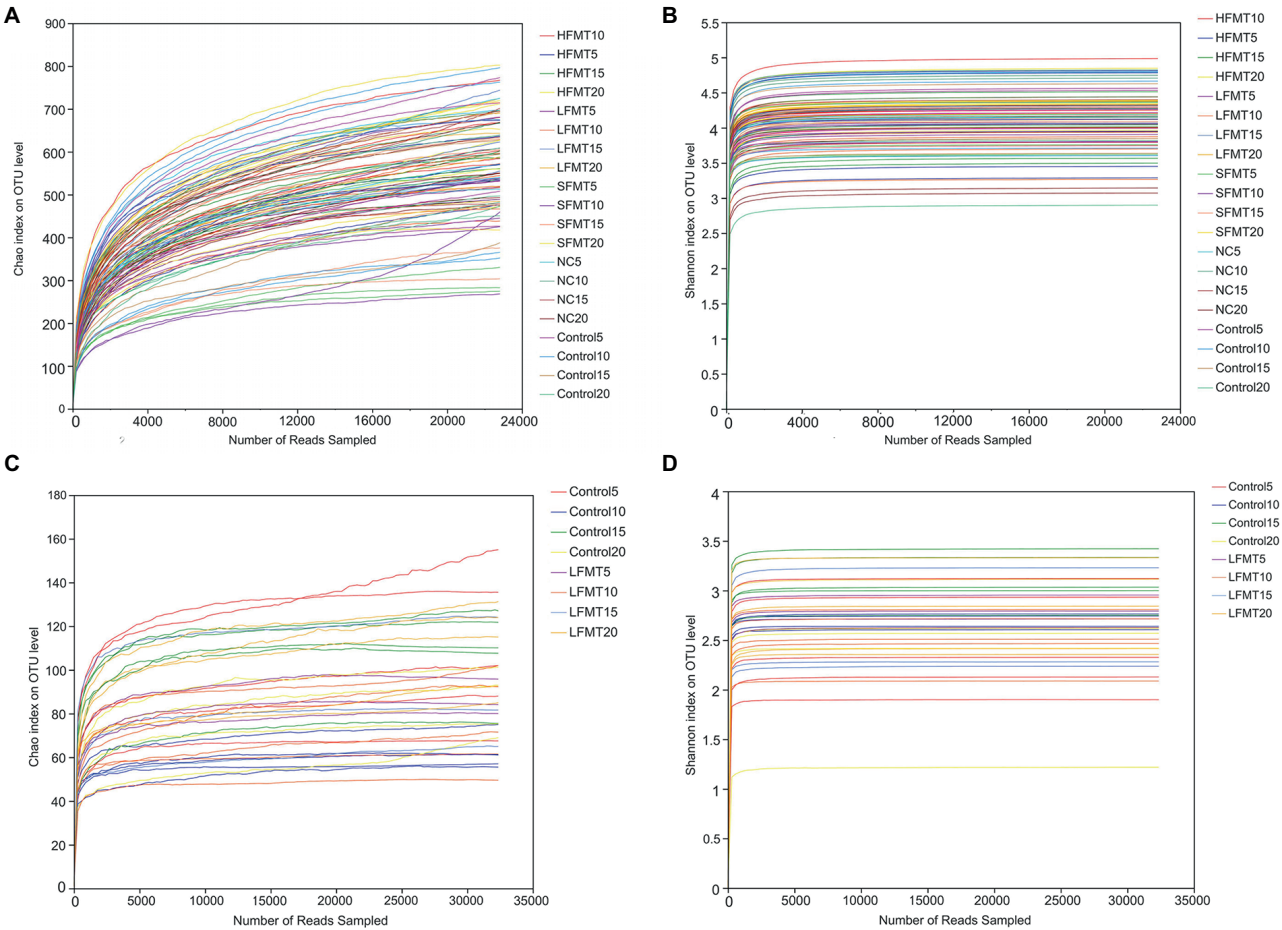
Sparsity curve analysis of stool samples. **(A)** Bacterial dilution curve based on Chao index. **(B)** Bacterial dilution curve based on Shannon index. **(C)** Fungal dilution curve based on Chao index. **(D)** Fungal dilution curve based on Shannon index. The x-axis represents the amount of randomly sampled sequencing data; the y-axis represents the number or diversity index of observed species. Each curve shown in the graph with a different color represents a sample.

As shown in Figure 2, with the increase of reads, the dilution curves of the Chao and Shannon indices of the fecal microbiota flattened, additionally, the community coverage of all samples was higher than 99% (Table 2), indicating that the sequencing data reached saturation, which covered most of the species in the group community.

**Table 2:** Effects of different FMT treatments on the alpha diversity index of fecal bacteria in weaned Holstein calves.

	Experimental treatment					SEM^1^	*p*						
	NC	Control	LFMT	HFMT	SFMT		Trt	Time	Trt × Time	Control × NC	Control × LFMT	Control × HFMT	Control × SFMT
Coverage index													
5 days	0.995^b^	0.996^a,b^	0.996^a^	0.995^b^	0.995^a^	0.00	< 0.01	0.38	0.28	0.78	0.64	0.75	0.13
10 days	0.994	0.997	0.996	0.995	0.998	0.00				0.79	0.82	0.66	0.15
15 days	0.994	0.997	0.993	0.995	0.996	0.00				0.18	0.39	0.79	0.65
20 days	0.995	0.996	0.993	0.996	0.994	0.00				0.42	0.11	0.17	0.28
ACE index													
5 days	595.68^a^	572.92^a,b^	526.09^a,b^	584.94^a^	392.89^b^	24.26	< 0.01	0.63	0.08	0.77	0.51	0.87	0.08
10 days	637.48^a^	585.88^a,b^	508.82^b^	633.58^a^	434.05^b^	27.86				0.62	0.48	0.66	0.23
15 days	618.08	537.73	592.47	546.12	500.07	20.31				0.25	0.49	0.89	0.65
20 days	532.24^a,b^	496.76^b^	620.41^a^	607.02^a^	614.05^a,b^	20.53				0.38	< 0.04	< 0.05	0.18
Chao index													
5 days	608.96^a,b^	591.66^a,b^	535.87^a,b^	577.94^a^	397.20^b^	25.17	< 0.01	0.64	0.11	0.83	0.45	0.86	0.06
10 days	653.12^a^	590.03^a,b^	512.96^b^	638.63^a^	455.85^b^	27.77				0.54	0.48	0.66	0.28
15 days	644.76	555.58	600.158	554.83	485.96	21.78				0.23	0.53	0.99	0.43
20 days	540.58^a,b^	502.04^b^	626.00^a^	627.93^a^	610.87^a,b^	21.19				0.34	0.04	0.03	0.20
Shannon index													
5 days	4.32^a^	4.21^a,b^	4.01^a,b^	4.09^a,b^	3.81^b^	0.08	0.47	0.68	< 0.01	0.58	0.27	0.75	0.06
10 days	4.61^a^	4.31^a,b^	3.71^b^	4.39^a,b^	3.99^b^	0.09				0.36	0.08	0.82	0.27
15 days	4.30^a^	4.05^a,b^	4.10^b^	3.87^b^	4.12^a,b^	0.06				0.20	0.82	0.49	0.76
20 days	3.74	3.92	4.35	4.24	4.38	0.09				0.65	0.16	0.30	0.16
Simpson index													
5 days	0.041^b^	0.089^a,b^	0.061^a^	0.031^a,b^	0.045^a^	0.00	0.87	0.54	0.04	0.30	0.57	0.84	0.96
10 days	0.015^b,c^	0.017^b,c^	0.055^a^	0.015^c^	0.050^a,b^	0.00				0.84	0.01	0.78	0.15
15 days	0.023^b^	0.060^a^	0.073^a^	0.141^a,b^	0.037^a,b^	0.01				0.07	0.67	0.44	0.54
20 days	0.031	0.246	0.037	0.037	0.033	0.01				0.72	0.40	0.43	0.38

Experimental treatments: NC, no supplementation; Control, normal saline; LFMT, 1 × 10^8^ CFU/ml bacterial solution/calf/time; HFMT, 1 × 10^9^ CFU/ml bacterial solution/calf/time; SFMT, sterilized bacterial solution; ^a,b,c^In the same row, values with different letter superscripts mean significant difference (*p* < 0.05).

1 SEM, standard error of mean.

### Diversity analysis of fecal microbiota

The Alpha diversity indices of the fecal microorganisms of weaned calves are shown in Tables 2, 3. The coverage index of each sample was > 0.993, indicating that sequencing results reflected the real compositions of the samples. The bacterial Alpha diversity index analysis showed that there was no significant difference in the bacterial diversity between the NC and Control group at all four time points. We found that the abundance-based coverage estimator (ACE) indices of the FMT treatment groups (LFMT, HFMT, and SFMT) were lower than those of the NC group at days 5, 10, and 15 after weaning (Table 2), whereas they were higher than those of the NC group at day 20 after weaning. The Chao1 indices of the FMT treatment groups were lower than those of the NC and Control groups at day 5 after weaning, and higher than that of the Control group at day 20 after weaning. The Shannon index in the LFMT group was significantly lower than that in the NC group (*p* < 0.05) at days 10 and 15 after weaning, but higher (*p* < 0.05) at day 20 after weaning. In addition, treatment had a significant effect on bacterial Chao and ACE indices, whereas the interaction between treatment and time had a significant effect on bacterial Shannon and Simpson indices. The fungal Alpha diversity index showed that FMT treatment had no significant effect on the fungal ACE, Chao, Shannon, and Simpson indices at the same time points (Table 3).

**Table 3:** Effects of LFMT treatment on the alpha diversity index of fecal fungi in weaned Holstein calves.

	Experimental treatments		*p*
	Control	LFMT	
Coverage index			
5 days	0.9999 ± 0.0000	0.9998 ± 0.0000	0.162
10 days	0.9998 ± 0.0000	0.9999 ± 0.0000	0.355
15 days	0.9999 ± 0.0000	0.9998 ± 0.0000	0.811
20 days	0.9997 ± 0.0000	0.9998 ± 0.0000	0.258
ACE index			
5 days	87.47 ± 13.79	105.88 ± 5.12	0.364
10 days	77.37 ± 3.78	64.71 ± 9.36	0.245
15 days	91.70 ± 8.20	108.37 ± 15.48	0.33
20 days	107.46 ± 8.90	81.20 ± 8.49	0.072
Chao index			
5 days	86.63 ± 15.86	109.60 ± 4.71	0.325
10 days	75.35 ± 3.45	61.95 ± 9.67	0.228
15 days	90.20 ± 8.97	108.39 ± 17.57	0.34
20 days	109.55 ± 7.51	84.65 ± 8.95	0.079
Shannon index			
5 days	2.79 ± 0.23	2.48 ± 0.09	0.371
10 days	2.52 ± 0.03	2.69 ± 0.12	0.208
15 days	2.58 ± 0.12	3.11 ± 0.32	0.116
20 days	2.81 ± 0.33	2.21 ± 0.19	0.139
Simpson index			
5 days	0.11 ± 0.04	0.18 ± 0.01	0.271
10 days	0.16 ± 0.01	0.14 ± 0.03	0.529
15 days	0.17 ± 0.01	0.09 ± 0.05	0.071
20 days	0.15 ± 0.12	0.26 ± 0.03	0.363

Experimental treatments: Control, normal saline; LFMT, 1 × 10^8^ CFU/ml bacterial solution/calf/time; The data at 4 time points after weaning are shown as mean ± SEM.

**Figure 3:**
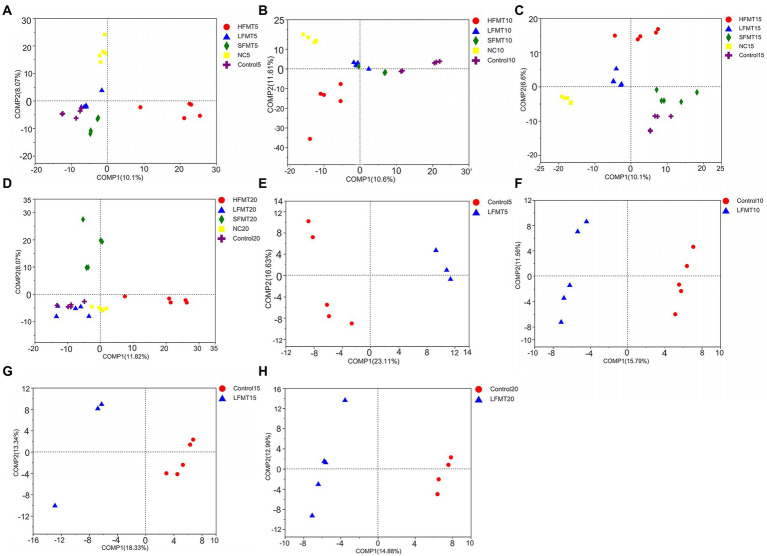
PLS-DA score plot to differentiate fecal microbial communities between the FMT and untreated groups at four time points after weaning. **(A)** Fecal bacterial community among groups at 5 days after weaning. **(B)** Fecal bacterial community among groups at 10 days after weaning. **(C)** Fecal bacterial community among groups at 15 days after weaning. **(D)** Fecal bacterial community among groups at 20 days after weaning. **(E)** Fecal fungal community among groups at 5 days after weaning. **(F)** Fecal fungal community among groups at 10 days after weaning. **(G)** Fecal fungal community among groups at 15 days after weaning. **(H)** Fecal fungal community among groups 20 days after weaning.

The similarity analysis of bacterial and fungal communities in fecal samples at different time points after weaning of calves showed (Table 4) that bacterial communities clustered well in different sample treatment groups at days 5 (Bray–Curtis ANOSIM = 0.2958), 10 (Bray–Curtis ANOSIM = 0.3164), 15 (Bray–Curtis ANOSIM = 0.4168), and 20 (Bray–Curtis ANOSIM = 0.3629) after weaning, with differences between groups being greater than those within groups. The fungal community clustering results at days 5 (Bray–Curtis ANOSIM = 0.7949), 10 (Bray–Curtis ANOSIM = 0.712), and 20 (Bray–Curtis ANOSIM = 0.3938) after weaning also showed that the differences between groups were significantly greater than those within groups. The results of the PLS-DA (Figure 3) showed that the samples from calves that received different concentrations of fecal microbiota and those from the NC and Control groups could be clearly distinguished and clustered. Further, the FMT treatment had a significant effect on the composition of intestinal microbiota at days 10 and 15 after weaning. In addition, we selected Control group and LFMT treatment group with low diarrhea rate for PLS-DA analysis, and found that LFMT treatment also had a significant effect on fungal community composition. It can also be seen from the dispersion of the distribution of sample points in the PLS-DA that there are also differences among individual gut microbes of different calves.

**Table 4:** Similarity analysis of fecal bacteria and fungi in weaned Holstein calves with different concentrations of FMT (ANOSIM).

Variable^1^	Bray-Curtis ANOSIM	*p*-value	Binary-Jaccard ANOSIM	*p*-value^2^
Bacteria				
5 days	0.2958	0.001	0.3363	0.001
10 days	0.3164	0.001	0.295	0.001
15 days	0.4168	0.001	0.307	0.002
20 days	0.3629	0.001	0.4138	0.001
Fungi				
5 days	0.7949	0.018	1	0.018
10 days	0.712	0.004	0.492	0.004
15 days	0.1692	NS	0.3026	NS
20 days	0.3938	0.018	0.2813	0.035

1 Calves treated with different concentrations of FMT before weaning on the 5th, 10th, 15th and 20th days after weaning.

2 NS, no significant; (*p* > 0.05).

### Analysis of fecal microbial community composition

**Figure 4:**
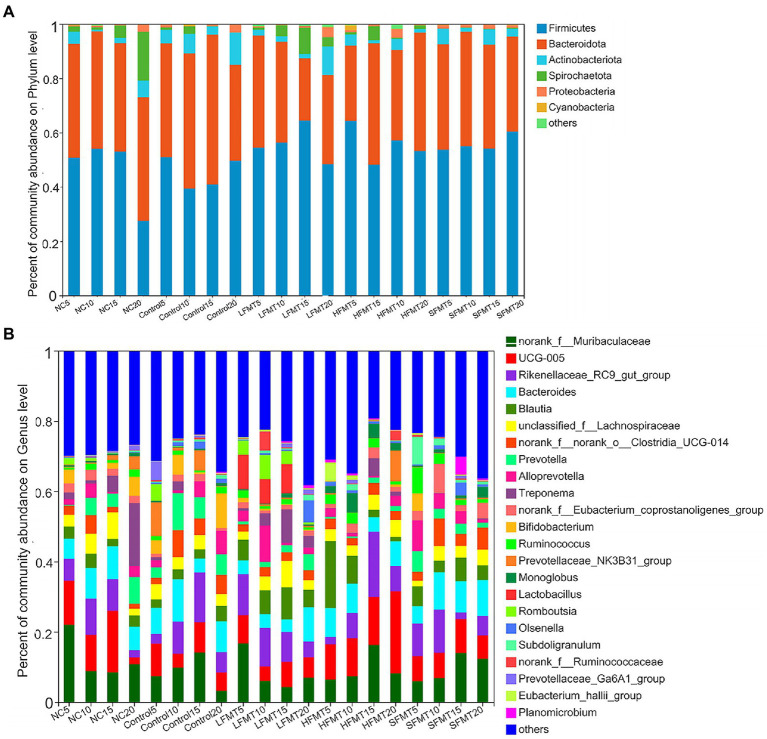
Comparison of fecal bacterial microbiota composition of FMT and untreated Holstein calves at four time points after weaning. **(A)** Flat relative abundance of bacterial microbiota in phylum level. **(B)** Relative abundance of bacterial microbiota composition at the genus level.

To explore the effect of FMT on fecal microorganisms of post-weaning calves, we compared the compositions of fecal bacterial community in the FMT treatment groups (LFMT group, HFMT group and SFMT group) and NC and Control groups. The relative abundance and composition of the 5 groups of samples at the phylum level at four time points after weaning are shown in Supplementary Table 4; Figure 4A. At the phylum level, gut bacteria were dominated by *Firmicutes* and *Bacteroidota*, followed by *Actinobacteriota*. At days 5 and 10 after weaning, abundances of *Firmicutes* in the FMT treatment groups were higher than those in the untreated groups, and the abundance in the LFMT group was significantly higher than that of the Control group at day 15 and that of the NC group at day 20 (*p* < 0.05). The abundance of *Bacteroidota* in the FMT-treated group was lower than that in the untreated group over days 0–15 after weaning. In addition, treatment and the interaction of time and treatment had significant effects on *Spirochaetota* abundance (*p* < 0.05). At the genus level, the fecal bacterial microbiota composition is shown in Supplementary Table 5; Figure 4B. *Norank*_*f_Muribaculaceae* and *UCG-005* were the main genera, followed by *Rikenellaceae_RC9_gut_group*, *Bacteroides*, *Blautia*, *unclassified_f_Lachnospiraceae*, *norank_f_norank_o_Clostridia_UCG-014*, *Prevotella*, *Alloprevotella*, *Treponema*, *norank_f__Eubacterium_coprostanoligenes_group*, *Bifidobacterium_coprostanoligenes_group*, *Bifidobacterium*, and *Ruminococcus*. The relative abundance of *Blautia* in the LFMT group was higher than that in the NC and Control groups at four time points after weaning. *Lactobacillus* was not detected in the Control group, at 5 days after weaning in the NC group or HFMT group, but a small amount was detected in the SFMT group at 15 days after weaning, and its relative abundance was higher in the LFMT group*. Norank_f_Muribaculaceae*, *Rikenellaceae_RC9_gut_group*, *Bacteroides*, *Blautia*, *unclassified_f_Lachnospiraceae*, *Alloprevotella*, and *Treponema* were not significantly affected by the treatment. The interaction of treatment with time had a significant effect on the abundance of *Rikenellaceae_RC9_gut_group*, *norank_f_norank_o_Clostridia_UCG-014*, *Prevotella*, *Ruminococcus*, and *Christensenellaceae_R-7_group*.

**Figure 5:**
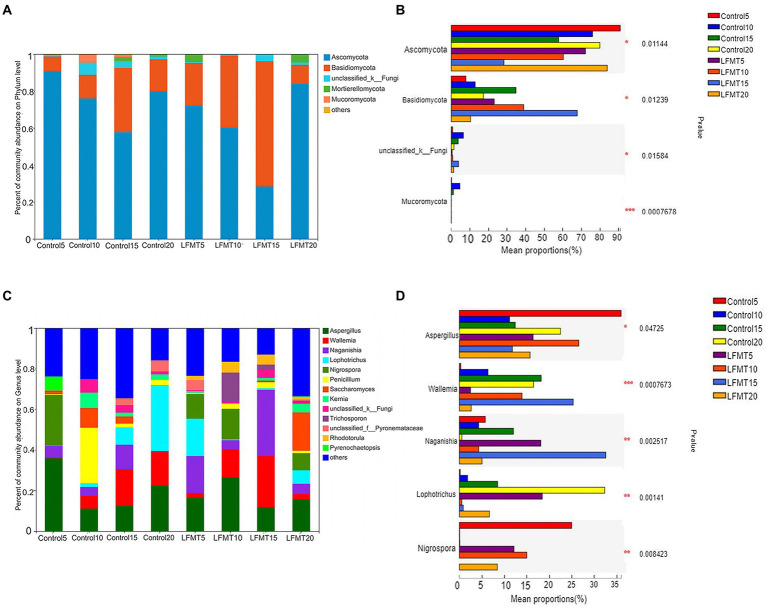
Comparison of fecal fungal microbiota composition of Holstein dairy calves in the LFMT and Control groups at four time points after weaning. **(A)** Relative abundance of fungal microbiota composition at the phylum level. **(B)** Significantly different phyla were identified using the Kruskal–Wallis *H*-test. **(C)** Relative abundance of fungal microbiota composition at the genus level. **(D)** Significantly different genera were identified using the Kruskal–Wallis *H*-test. ^*^p < 0.05, ^**^p < 0.01 and ^***^p ≤ 0.001.

We analyzed the fungal community composition of Control group and LFMT group with low diarrhea rate, and found that *Ascomycota* and *Basidiomycota* were the main phyla of fecal fungal colonies after weaning of calves, followed by *unclassified_k_fungi*, *Mortierellomycota*, and *Mucoromycota* (Figure 5A). The relative abundance of *Ascomycota* in the LFMT group was lower than that in the Control group, whereas the relative abundance of *Basidiomycota* was higher in the fecal samples at days 5, 10, and 15 after weaning. Relative abundances of *Ascomycota*, *Basidiomycota*, and *Mucoromycota* were significantly different between groups (*p* < 0.05, Figure 5B). *Aspergillus* and *Wallemia* were the main genera, followed by *Naganishia*, *Lophotrichus*, *Nigrospora*, *Penicillium*, *Saccharomyces*, *Kernia, unclassified_k_Fungi*, and *Trichosporon*. Relative abundances of *Wallemia* and *Naganishia* in the LFMT group were higher than those in the Control group at each time point. The fecal samples were then analyzed for the significance of differences between the dominant fungi genera. The results showed that at the genus level, there were significantly different (*p* < 0.05) or extremely significantly different (*p* < 0.01) among the top 5 genera at different time points (Kruskal–Wallis *H*-test).

**Figure 6:**
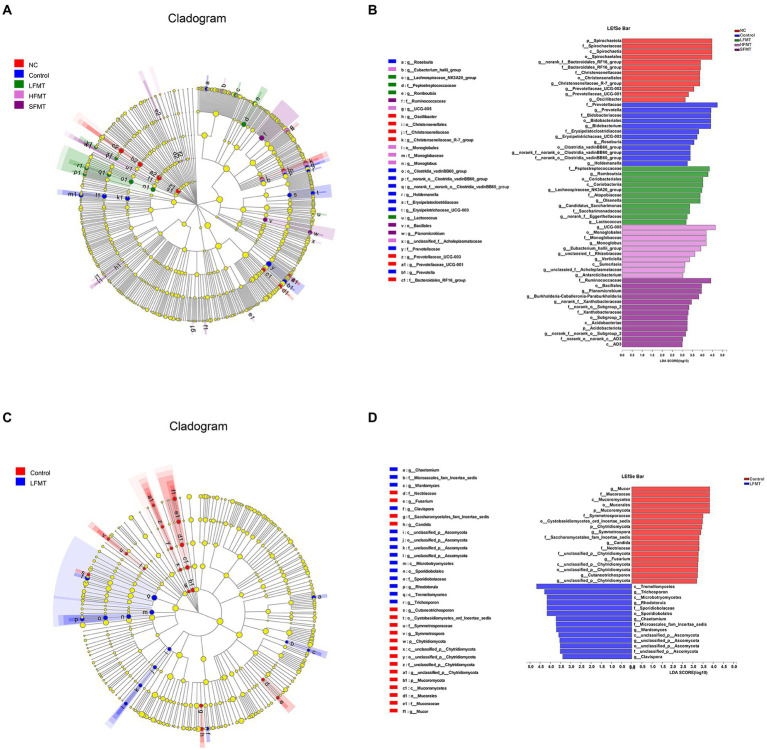
LEfSe analysis comparing the differential microbiota among groups. Clade diagram showing the phylogenetic distribution of microbial lineages across different FMT groups. Differences in abundance between groups were assessed using LDA scores. **(A)** Phylogenetic distribution of bacterial microbiota associated with FMT group and untreated group taxa. **(B)** LDA > 3 differential bacteria in bacterial communities associated with FMT and untreated groups. **(C)** Phylogenetic distribution of fungal microbiota associated with FMT group and untreated group taxa. **(D)** Differential fungi with LDA > 3 in the fungal community associated with the FMT group and the untreated group. The colored circles from the inside to the outside of the LEfSe analysis cladogram represent different taxonomic levels (phylum, class, order, family, genus). The diameter of each circle is proportional to the abundance of the population. The yellow circles in the cladogram represent taxa that are not significantly different. LDA scores > 3 were considered statistically significant. Different colored areas represent different components.

For further assessment of the differences in fecal microbiota among the five groups of samples, linear discriminant analysis (LDA) was carried out for fecal microorganisms at different taxonomic levels with LDA > 3 (*p* < 0.05) as the critical value (Figure 6). It was found that each treatment group had some microorganisms that were significantly enriched on the phylum and genus levels. Bacterial groups were analyzed, and *norank_f_Bacteroidales_RF16_group*, *Christensenellaceae_R-7_group*, and *Prevotellaceae_UCG-003* were significantly enriched in the NC group. *Prevotella*, *Bifidobacterium*, *Erysipelotrichaceae_UCG-003*, and *Roseburia* were more abundant in the Control group. In the LFMT group, *Romboutsia*, *Olsenella*, *Lachnospiraceae_NK3A20_group*, *Candidatus_Saccharimonas*, *norank_f_Eggerthellaceae*, and *Lactococcus* were significantly more abundant than in other groups. In the HFMT group, *UCG-005*, *Monoglobus*, *Eubacterium_hallii_group*, and several others were significantly enriched. In the SFMT group, *Planomicrobium*, *Burkholderia–Caballeronia–Paraburkholderia, norank_f_Xanthobacteraceae*, and others were more abundant. Fungal taxonomic analysis showed that in the Control group, genus *Mucor* was significantly enriched, followed by *Symmetrospora*. *Trichosporon* was more abundant in the LFMT group, followed by *Rhodotorula*, *Wardomyces*, and several other genera.

### Fecal microbial network analysis

**Figure 7:**
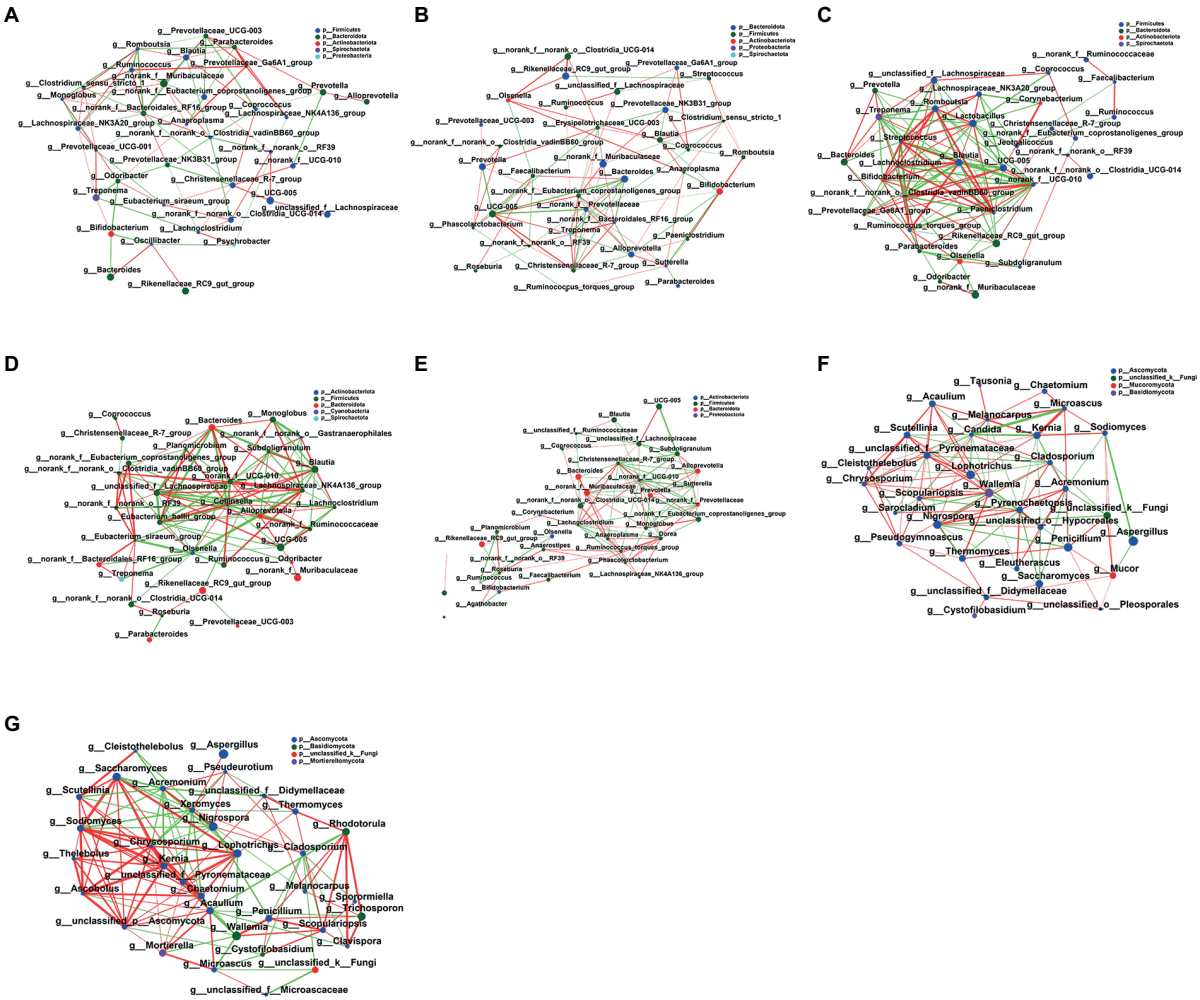
Interaction network diagram of fecal microbiota in each group. **(A)** The network diagram of the interaction between the fecal bacterial microbiota in the NC group. **(B)** Interaction network diagram of fecal bacterial microbiota in the Control group. **(C)** Interaction network diagram of fecal bacterial microbiota in LFMT group. **(D)** Interaction network diagram of fecal bacterial microbiota in HFMT group. **(E)** Interaction network diagram of fecal bacterial microbiota in SFMT group. **(F)** The network diagram of the interaction between fecal fungi in the Control group. **(G)** Interaction network diagram of fecal mycobiota in the LFMT group. The size of the nodes in the figure represents the abundance of species, and different colors represent different species; the color of the connection line represents positive and negative correlation, red represents positive correlation, and green represents negative correlation; the thickness of the line represents the magnitude of the correlation coefficient, the line Thicker lines indicate higher correlations between species; more lines indicate closer connections between the species and other species.

To explore the effect of FMT on fecal microbial interactions in early weaned calves, we analyzed microbial networks for interactions between the top 35 bacterial and fungal communities as judged by the total abundance at the taxonomic level. Figure 7; Supplementary Table 6 illustrates interaction networks of fecal microorganisms in bacterial NC, Control, LFMT, HFMT, SFMT groups and fungal Control and LFMT groups. FMT treatment groups edge_ Num and average node connectivity were higher than NC group and control group. We analyzed the Control and LFMT groups that had higher network complexity and found that in the Control group, bacteria *norank_f_Prevotellaceae* and *Christensenellaceae_R-7_group* (degree = 11) and fungus *Wallemia* (degree = 15) had the largest number of connected nodes, followed by *norank_f__Eubacterium_coprostanoligenes_group*, *UCG-005* (degree = 10), *Treponema* (degree = 9), *Pyrenochaetopsis*, and *Candida* (degree = 14). However, the more abundant genera in bacteria, such as *Rikenellaceae_RC9_gut_group* (degree = 3), and the most abundant fungal genus *Aspergillus* (degree = 3) had very low node connectivity. Furthermore, we found 55 positive and 43 negative correlations in the bacterial network lines of the Control group, and 78 positive and 50 negative correlations in the fungal network lines. In the LFMT group, *norank_f__norank_o__ Clostridia_vadinBB60_group*, *Streptococcus*, *Chrysosporium* (degree = 16 for all) had the largest number of connected nodes, followed by *Lactobacillus*, *norank_f__UCG-010*, *Paeniclostridium*, *Romboutsia* (degree = 15), *Ruminococcus_torques_group*, *Chaetomium* (degree = 14), *UCG-005*, *Blautia*, *Kernia*, *Sodiomyces*, *Acaulium*, *Acremonium* (degree = 13). In contrast, for other genera with higher abundance, such as *norank_f_Muribaculaceae* (degree = 2) or *Aspergillus* (degree = 1), node connectivity was very low, whereas *Rikenellaceae_RC9_gut_group* (degree = 13) node connectivity was improved compared with the Control group. After the FMT treatment, there were 60 positive correlations and 70 negative correlations in bacterial network lines in the LFMT group, 91 positive correlations and 48 negative correlations in the fungal network, and the correlations were different compared to those in the Control group.

### Functional prediction

**Figure 8:**
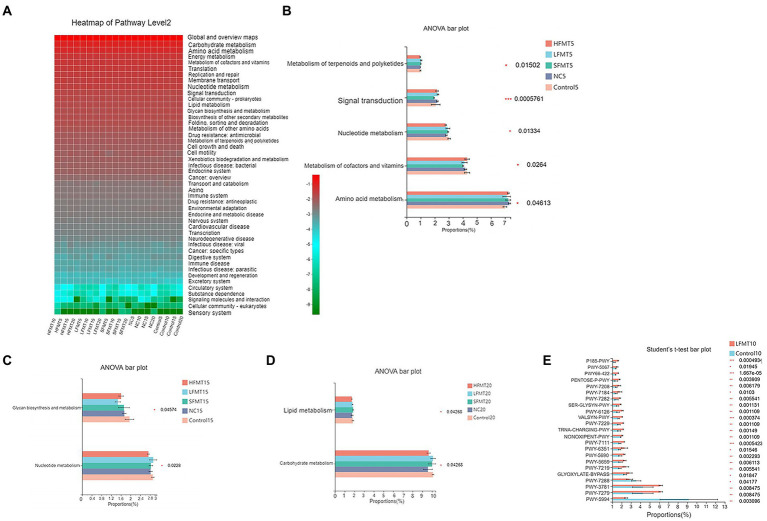
Functional prediction analysis of bacterial 16 s rRNA and ITS gene sequences based on KEGG database and MetaCyc database, respectively. **(A)** Relative enrichment of KEGG Level 2 in different treatment groups. **(B)** One-way ANOVA of KEGG Level 2 levels in each treatment group 5 days after weaning. **(C)** One-way ANOVA of KEGG Level 2 in each treatment group 15 days after weaning. **(D)** One-way ANOVA of KEGG Level 2 in each treatment group 20 days after weaning. **(E)** Student’s *t*-test was performed on the functional pathways of each treatment group based on MetaCyc database 10 days after weaning.PWY-7279 [aerobic respiration II (cytochrome c; yeast)], PWY-3781 (aerobic respiration I (cytochrome c)), GLYOXYLATE-BYPASS (glyoxylate cycle), PWY-5994 (palmitate biosynthesis I (animals and fungi)), PWY-7282 (4-amino-2-methyl-5-phosphomethylpyrimidine biosynthesis (yeast)), PWY-7288 (fatty acid &beta;-oxidation (peroxisome, yeast)), PWY-7111 (pyruvate fermentation to isobutanol (engineered)), PWY-7219 (adenosine ribonucleotides *de novo* biosynthesis), PWY-5690 (TCA cycle II (plants and fungi)), PWY-6351 (D-myo-inositol (1,4,5)-trisphosphate biosynthesis), PWY-5659 (GDP-mannose biosynthesis), SER-GLYSYN-PWY (superpathway of L-serine and glycine biosynthesis I), NONOXIPENT-PWY (pentose phosphate pathway (non-oxidative branch)), TRNA-CHARGING-PWY (tRNA charging), PWY-7229 (superpathway of adenosine nucleotides *de novo* biosynthesis I), PWY-7184 (pyrimidine deoxyribonucleotides *de novo* biosynthesis I), PENTOSE-P-PWY (pentose phosphate pathway), PWY-7208 (superpathway of pyrimidine nucleobases salvage). The abscissa represents the relative abundance of features in the sample, the ordinate represents the name of the feature, and different colors represent different groups. The right side is the corrected *p* value, ^*^0.01 < *p* < 0.05, ^**^0.001 < *p* < 0.01, ^***^p ≤ 0.001.

To predict changes in the metagenome functional content following FMT based on 16S rRNA and ITS sequences, we used PICRUSt2 software. The results showed that at level 2 of the Kyoto Encyclopedia of Genes and Genomes (KEGG) pathway, bacterial functional characteristics were mainly enriched in metabolic pathways such as carbohydrate metabolism, amino acid metabolism, and energy metabolism (Figure 8A). One-way analysis of variance was performed on functional pathways with relative abundance greater than 1%, and differences in functional gene metabolic pathways in bacterial microbiota between different groups were observed. Five gene functional pathways, including amino acid metabolism, metabolism of cofactors and vitamins, nucleoside metabolism, signal transduction, and metabolism of terpenoids and polyketides were different between groups at day 5 after weaning (Figure 8B). At day 10 after weaning, only the folding, sorting, and degradation functional pathways were significantly different (*p* < 0.05). There were significant differences in two functional pathways, nucleotide metabolism and glycan biosynthesis and metabolism, at day 15 after weaning (Figure 8C). There were significant differences in carbohydrate metabolism and lipid metabolism between groups at day 20 after weaning (Figure 8D). Analysis of the top 30 MetaCyc pathways in the relative abundance of fungal genomes showed that PWY-922 mevalonate pathway I was enriched in the LFMT group at day 5 after weaning (*p* < 0.05). At day 10 after weaning, there were 23 significantly different functional pathways in the Control and LFMT groups (Figure 8E; *p* < 0.05), and the differential functional pathways were mainly abundant in the LFMT group. At days 15 and 20 after weaning, no significant differences in gene functional pathways were observed.

## Discussion

The structure of gut microbiota is influenced not only by genetic factors, but is also susceptible to population effects of diet, health status, living environment, and environmental symbiotic microorganisms (Bang et al., 2019). At weaning, gut microbiota dynamically changes and is easily influenced by the surrounding environment (Li et al., 2020; Zhang et al., 2021). Therefore, performing FMT during this period provides an opportunity to improve the health of weaned calves by rebuilding the gut microbiota structure.

Recently, FMT was shown to effectively improve the symptoms of diarrhea in calves before weaning (Kim et al., 2021). Our present results show that FMT before weaning had a good preventive effect on diarrhea after weaning in calves, which is consistent with a previous report about FMT efficacy in improving diarrhea in piglets after weaning (Su et al., 2021). The diarrhea rate and diarrhea index of the FMT groups were lower than those in the untreated groups at days 1–10 after weaning, which may be because FMT improved the structure of intestinal microbiota in the post-weaning period, reduced the imbalance of intestinal microbiota, and alleviated diarrhea symptoms in calves. In addition, the total diarrhea rate in the LFMT group was lower than that in the HFMT group, which was similar to the results of a previous study of the effect of FMT on the diarrhea rate in weaned piglets (Hu et al., 2018b), suggesting appropriate microbial doses need to be used for gut microbial intervention as treatment with high concentrations of microbes may affect tolerance and disrupt intestinal balance in the recipient (Tang, 2019). Interestingly, the diarrhea rate also decreased in the SFMT group that received autoclaved microbial preparations. Some scholars pointed out that FMT grafts include not only living and dead bacteria, but also intestine cells and bacterial metabolites (Bojanova and Bordenstein, 2016). Whether living bacteria or bacteria that have been sterilized may not be the only factor affecting the biological changes of the receptor, which also explains that the diarrhea rate of SFMT has decreased in our study, but it is different from the results of LFMT and HFMT. Similar results appeared in previous reports (Muscato et al., 2002). At present, for most diseases, the effective mechanism of FMT is not clear, which also needs further research.

The Alpha diversity analysis of fecal bacterial microbiota of weaned calves showed that the ACE, Chao, and Shannon indices in the FMT treatment groups were lower than those in the NC and Control groups at the beginning of weaning, and the Simpson index was higher than those in the NC and Control groups. However, this situation was reversed at day 20 after weaning. It is possible that fecal microorganisms of the donor had successfully colonized the recipient’s intestine, which increased the richness and diversity of the recipient’s fecal bacterial microbiota. However, despite the fact that the treatment had a significant impact on the fecal bacterial microbiota richness (*p* < 0.05), it did not significantly affect the diversity. This observation is consistent with the results of the study on the adaptation of intestinal microbiota during fecal bacteria transplantation in human infants (Gu et al., 2016). In addition, FMT had a weaker effect on the fungal microbiota than on bacteria, as FMT did not significantly change the fungal Alpha diversity index.

FMT was demonstrated to significantly alter the composition of calf gut microbiota (Wu, 2018), which was also confirmed in our microbial community similarity analysis and PLS-DA. *Firmicutes* and *Bacteroidota* were the main phyla of the fecal bacterial microbiota of post-weaning calves, which is consistent with previous studies showing the predominance of these two phyla in human fecal microbes and other mammalian gastrointestinal microbes (Costea et al., 2017; Liu et al., 2019). Similarly, the abundance of *Firmicutes* was high in donor yaks in our study. At different time points after weaning, the abundance of *Firmicutes* in the FMT treatment groups was higher than that in the NC and Control groups, indicating that FMT increased the relative abundance of *Firmicutes*. It has been shown previously that fecal microbiota of the recipient tends to become closer to that of the donor (Cao et al., 2018; Smillie et al., 2018), however, the abundance of *Firmicutes* varied in different FMT groups. For example, the abundance of *Firmicutes* in the HFMT group was the highest at day 5 after weaning, whereas that in the SFMT group was the highest at day 20 after weaning, which may be related to the tolerance of the recipient to different concentrations of bacterial suspension or colonization of the recipient by microorganisms from the donor fecal bacterial suspension (Tang, 2019). Interestingly, the abundance of *Bacteroidota* was lower in the LFMT group compared to that in untreated groups, which is consistent with previous studies showing that combined supplementation with sodium humate and glutamine alleviated diarrhea incidence in weaned calves by altering gut microbiota and metabolites (Wang et al., 2021). The interaction between treatment and time had a significant effect on *Spirochaetota*, and previous studies indicated that *Spirochaetota* abundantly express carbohydrate degradation genes (Zhou et al., 2020) and decompose cellulose, pectin, and phosphate (Matthias et al., 2011; Wang et al., 2021). The relative abundance of *Spirochaetota* was higher on days 10 and 15 after weaning in the LFMT treatment group and suggesting that the short chain fatty acids produced by carbohydrate degradation were higher than those in other treatment groups. Short chain fatty acids play an important role in improving intestinal function, resisting pathogenic microorganisms, regulating host immune system function and providing energy for host epithelial cells (Verbeke et al., 2015), which likely contributed to the alleviation of diarrhea in calves after weaning to a certain extent. However, interestingly, in the LEfSe analysis, *Spirochaetota* was a biomarker of the NC group. This finding could be related to the sudden increase in the relative abundance of *Spirochaetota* in two samples of the NC group at day 20 after weaning, which also shows that fecal microbiota was affected by many factors, and there were certain differences between individual calves held under the same drinking, feeding, and management conditions. In addition, the diarrhea rate of the NC group did not decrease significantly in 20 days after weaning, which may be related to the high abundance of *Spirochaetota*, because in some cases, the excessive production of short chain fatty acids may be unfavorable to the host (Serino, 2019), so how *Spirochaetota* interact with the host remains to be further studied.

*Blautia*, *Alloprevotella*, and *Lactobacillus* are closely associated with animal health. Earlier reports suggest that *Blautia* plays a role in biotransformation and interaction with other gut microbes that can maintain intestinal environmental balance and prevent inflammation by upregulating intestinal regulatory T cells and by short chain fatty acid production (Kim et al., 2014). In addition, bacteriocin produced by *Blautia* inhibited colonization of the intestine by pathogenic bacteria and affected the composition of intestinal microbiota (Liu et al., 2021a). Another study showed that *Blautia* abundance was significantly reduced in the cecal mucosal microbiota in patients with Crohn’s disease (Chen et al., 2014). In our study, we found that compared with abundances in the NC and Control groups, the abundance of *Blautia* in the LFMT group was higher at four time points after weaning. In addition, abundances of *Blautia* in the HFMT and SFMT groups were also higher at different time points. Thus, the low diarrhea rates in the FMT treatment groups could be related to the high abundance of *Blautia*. Some studies have pointed out that genus *Alloprevotella* was related to fiber digestion and generation of the anti-inflammatory effect. In addition, it was showed that weaning stress in piglets reduced the relative abundance of *Alloprevotella* compared to that in lactating piglets (Downes et al., 2013; Li et al., 2018). In this study, the relative abundance of *Alloprevotella* in the FMT treatment groups was higher than that in untreated groups at day 5 after weaning, whereas in the LFMT group, *Alloprevotella* abundance was the highest at day 10 after weaning. It is speculated that FMT treatment can alleviate weaning stress by increasing the relative abundance of *Alloprevotella*.

We observed that the number of core OTUs in each group remained basically stable. On the 5th and 10th days after weaning, the number of HFMT specific OTUs was higher than that in other treatment groups. With the extension of time after weaning, the number of HFMT specific OTUs decreased, while the number of LFMT specific OTUs increased. On the 20th day after weaning, the number of LFMT specific OTUs was higher than that in other treatment groups. It is speculated that FMT treatment eliminated or inhibited some of the original microbiota of the receptor and established a new microbiota structure. Moreover, different FMT treatments have different effects. The special OTU in LFMT treatment group is mainly *Lactobacillus*. It is well established that *Lactobacillus* species are the most common constituents of probiotic preparations (Kaźmierczak-Siedlecka et al., 2021). They increase the expression of tight junction protein (Fata et al., 2018; Sanders et al., 2019) and production of organic acids in the metabolic process, which can inhibit the reproduction and colonization of pathogenic bacteria (Aoudia et al., 2016; David et al., 2016). Addition of *Lactobacillus* reduced incidence of gastrointestinal infections and animal diarrhea (Azagra-Boronat et al., 2020; Dong et al., 2021), indicating that there may be an inverse relationship between *Lactobacillus* abundance and diarrhea. It has been noted that *Lactobacillus* species are common in calf intestines before weaning (Malmuthuge et al., 2019), and that their abundance decreases with age (Uyeno et al., 2010; Klein-Jöbstl et al., 2014). Our study shows that *Lactobacillus* species had low abundance or were not detected in the Control group after weaning, which is consistent with the previous research results (Xiang et al., 2020). Further, the relative abundance of *Lactobacillus* in the LFMT group was higher than in untreated groups, which showed that low concentration of FMT treatment increased the relative abundance of beneficial *Lactobacillus*.

*Ruminococcus* was one of the most effective bacteria in decomposing carbohydrates (Qin et al., 2021; Wu et al., 2021), which has the function of stabilizing intestinal barrier and reversing diarrhea (Samantha et al., 2018). On the 5th and 15th days after weaning, FMT treatment increased the relative abundance of *Ruminococcus* in rectal feces, which was conducive to the maintenance of calf intestinal health. *Romboutsia* can utilize mixtures of sugars and peptides to produce short chain fatty acids such as isobutanoic acid and acetic acid (Wang et al., 2015; Liu et al., 2022). Short chain fatty acids play an important role in the maintenance of intestinal mucosal barrier, the regulation of intestinal motility, and immune regulation (Feng et al., 2018). FMT treatment had a significant effect on the relative abundance of *Romboutsia*. Ten to twenty days after weaning, the relative abundance of *Romboutsia* in LFMT group was higher than that in NC and Control group, suggesting that *Romboutsia* may play an important role in maintaining intestinal health.

*Ascomycota* and *Basidiomycota* were common fecal fungal phyla in donors and recipients. The relative abundance of *Ascomycota* in the donor was much higher than that of *Basidiomycota*. We observed that relative abundances of *Ascomycota* in the FMT treatment groups were higher than LFMT group on days 5, 10, and 15 after weaning. Relative abundances of were also higher than that of the Control group, but this relationship was reversed at day 20 after weaning. It is likely that microbiota in the recipient calves showed different resistance and ability to coexist with the introduced donor species (Li et al., 2016). *Wallemia* and *Naganishia* were significantly dominant genera in the LFMT group at day 15 post-weaning. It has been shown that the presence of *Wallemia* is related to the concentration of glucose and fructose, as well as to the activity of β-D-glucosidase in piglet colonic digest, which may play an important role in the degradation of complex carbohydrates in piglet hindgut (Luo et al., 2021). In addition, *Naganishia* is the main yeast genus producing extracellular enzymes (Tan et al., 2018). Extracellular enzymes decompose macromolecular nutrients to promote metabolism in the body. Therefore, it is speculated that the existence of these two fungi can promote the food digestion and metabolism of calves.

The coexistence network topologies of the intestinal microbiota in the NC, Control, and FMT groups were different. The complexity of the intestinal microbiota coexistence network in the FMT groups was high, which may be caused by altered interactions between intestinal microorganisms after FMT treatment. We found that the LFMT group had the highest network complexity, and the composition of this complex network was mainly caused by the enhanced correlation between genera of the phylum *Firmicutes*. In addition, *Lactobacillus*, *Romboutsia*, and *Blautia*, whose presence positively correlates with animal health, were more abundant in the LFMT group, and their correlation with other bacterial genera also became more complex, which is consistent with the results of our previous study (Liu et al., 2021b). In addition, genera *Lactobacillus*, *Romboutsia*, and *Blautia* positively correlated with each other. The complexity of the fungal network was mainly caused by the changes in the relationship between *Ascomycota* genera. It has been pointed out that complex networks increase the resistance to pathogen invasion (Wei et al., 2015; William et al., 2018). In addition, in the LFMT group, the connectivity between bacteria and fungi was very high. When the body is invaded by pathogens or there is a lack of specific microbial niche, the adjacent niche supplements any gap, limiting the nutritional supply of any invading microorganisms and leading to the extinction of pathogens (Liu et al., 2021b). Therefore, the complex microbial networks in the FMT groups likely conferred stronger resistance to external influences.

Gut microbiota is involved in regulating various metabolic pathways closely related to the health of the host. In this study, PICRUSt2 software was used to predict bacterial and fungal gene functions based on KEGG and MetaCyc databases. It was found that carbohydrate metabolism and amino acid metabolism were the most enriched pathways in the KEGG level 2 functional pathway, indicating that fecal microorganisms were active in various physiological states. We found that different FMT treatments had distinct effects on fecal microbial functional pathways, such as increasing the relative abundance of the nucleotide metabolism pathway to different degrees at different time periods. Nucleotides have the functions of regulating immune function, promoting the development of gastrointestinal tract and reducing the incidence of diarrhea (Jang and Kim, 2019). We speculate that the enhancement of nucleotide metabolism has a positive effect on alleviating diarrhea in weaned calves by increasing the content of nucleotides in the intestine. PWY-922 mevalonate pathway I is an important metabolic pathway involved in the synthesis of isoprenoids, which are essential for cell growth and a variety of cellular processes (Buhaescu and Izzedine, 2007). This pathway was enriched in the LFMT group, presumably promoting growth and development of weaned calves. In addition, the LFMT treatment increased the relative abundance of 23 functional pathways, such as glycogen biosynthesis II, D-galactose degradation V, pentose phosphate pathway, superpathway of pyrimidine nucleobases salvage, aerobic respiration I, aerobic respiration II, glyoxylate cycle, and others. This suggests that LFMT intervention may promote related metabolic processes and cellular processes. It is speculated that LFMT treatment improves the anti stress level of animals by enhancing the metabolic function of some pathways. Overall, the FMT treatment improved the ability of gut microbes to stimulate adaptability of the host to environmental changes. A limitation of this study was that metagenomic sequencing of fecal microbes was not performed to provide more accurate and direct evidence for future functional studies of gut microbiota.

## Conclusion

Taken together, our findings suggest that FMT significantly affected fecal microbial composition of weaned calves, increasing the relative abundance of beneficial gut microbiota and enhancing the richness and diversity of bacterial microbiota. The FMT treatment increased the complexity of the fecal microbial network of weaned calves, presumably improving resistance to pathogens. In addition, the LFMT treatment was better than HFMT and SFMT in reducing the occurrence of calf weaning diarrhea, so the mechanism of action and optimal dosage of fecal microbiota need to be further explored in future studies. The results of this study may serve as a reference for the treatment and prevention of calf weaning diarrhea and, potentially, other intestinal diseases in ruminants. In addition, our study provided useful information for further research on effective and safe non-antibiotic alternatives for the prevention and treatment of calf diarrhea.

## Data availability statement

The datasets presented in this study can be found in online repositories. The names of the repository/repositories and accession number(s) can be found at: National Center for Biotechnology Information Sequence Read Archive database under accession number SRP370467.

## Ethics statement

The animal study was reviewed and approved by Bioethics Committee of the Shihezi University.

## Author contributions

YL and XL wrote the first draft of the manuscript. YW prepared the materials for this manuscript. WZ revised this manuscript. All authors contributed to the article and approved the submitted version.

## Funding

This research was supported by the Scientific and Technological Research Project in Key Fields of Xinjiang Production and Construction Corps (2018AB041), China.

## Conflict of interest

The authors declare that the research was conducted in the absence of any commercial or financial relationships that could be construed as a potential conflict of interest.

## Publisher’s note

All claims expressed in this article are solely those of the authors and do not necessarily represent those of their affiliated organizations, or those of the publisher, the editors and the reviewers. Any product that may be evaluated in this article, or claim that may be made by its manufacturer, is not guaranteed or endorsed by the publisher.

## Footnotes

^1^ https://github.com/OpenGene/fastp

^2^ http://www.cbcb.umd.edu/software/flash

^3^ http://drive5.com/uparse/

^4^ http://unite.ut.ee/index.php

^5^ http://drive5.com/usearch/manual/singletons.html

^6^ http://rdp.cme.msu.edu/

## References

[ref1] Aoudia N. Rieu A. Briandet R. Deschamps J. Chluba J. Jego G.. (2016). Biofilms of *Lactobacillus plantarum* and *Lactobacillus fermentum*: effect on stress responses, antagonistic effects on pathogen growth and immunomodulatory properties. Food Microbiol. 53, 51–59. doi: 10.1016/j.fm.2015.04.009, PMID: 26611169

[ref2] Azagra-Boronat I. Massot-Cladera M. Knipping K. Garssen J. Pérez-Cano F. (2020). Strain-specific probiotic properties of bifidobacteria and lactobacilli for the prevention of diarrhea caused by rotavirus in a preclinical model. Nutrients 12:498. doi: 10.3390/nu12020498, PMID: 32075234 PMC7071190

[ref3] Bang S. J. Lee E. S. Song E. J. Nam Y. D. Seo M. J. Kim H. J.. (2019). Effect of raw potato starch on the gut microbiome and metabolome in mice. Int. J. Biol. Macromol. 133, 37–43. doi: 10.1016/j.ijbiomac.2019.04.085, PMID: 30986463

[ref4] Bojanova D. P. Bordenstein S. R. (2016). Fecal transplants: what is being transferred? PLoS Biol. 14:e1002503. doi: 10.1371/journal.pbio.1002503, PMID: 27404502 PMC4942072

[ref5] Brown K. Uwiera R. Kalmokoff M. L. Brooks S. Inglis G. D. (2017). Antimicrobial growth promoter use in livestock: a requirement to understand their modes of action to develop effective alternatives. Int. J. Antimicrob. Agents 49, 12–24. doi: 10.1016/j.ijantimicag.2016.08.006, PMID: 27717740

[ref6] Buhaescu I. Izzedine H. (2007). Mevalonate pathway: a review of clinical and therapeutical implications. Clin. Biochem. 40, 575–584. doi: 10.1016/j.clinbiochem.2007.03.016, PMID: 17467679

[ref7] Cammarota G. Ianiro G. Tilg H. Rajilić-Stojanović M. Kump P. Satokari R.. (2017). European consensus conference on faecal microbiota transplantation in clinical practice. Gut 66, 569–580. doi: 10.1136/gutjnl-2016-313017, PMID: 28087657 PMC5529972

[ref8] Cao Y. T. Zhang B. J. Wu Y. Y. Wang Q. Z. Wang J. Shen F. F. (2018). The value of fecal microbiota transplantation in the treatment of ulcerative colitis patients: a systematic review and meta-analysis. Gastroenterol. Res. Pract. 2018:5480961. doi: 10.1155/2018/5480961, PMID: 29849592 PMC5903331

[ref9] Chen L. P. Wang W. Zhou R. Ng S. C. Li J. Huang M. F.. (2014). Characteristics of fecal and mucosa-associated microbiota in Chinese patients with inflammatory bowel disease. Medicine 93:e51. doi: 10.1097/MD.0000000000000051, PMID: 25121355 PMC4602441

[ref10] Chen S. F. Zhou Y. Q. Chen Y. R. Gu J. (2018). Fastp: an ultra-fast all-in-one FASTQ preprocessor. Bioinformatics 34, i884–i890. doi: 10.1101/274100, PMID: 30423086 PMC6129281

[ref11] Clark E. O. Deller A. N. Harrelson P. Harrelson F. W. (2016). Effects of two-stage weaning duration on beef cattle growth and vocalizations. J. Anim. Sci. 95:58. doi: 10.2527/ssasas2017.0118

[ref12] Costea P. I. Hildebrand F. Manimozhiyan A. Bckhed F. Bork P. (2017). Enterotypes in the landscape of gut microbial community composition. Nat. Microbiol. 3, 8–16. doi: 10.1038/s41564-017-0072-8, PMID: 29255284 PMC5832044

[ref13] David R. C. Patricia R. M. Abelardo M. Miguel G. De L. Nuria S. (2016). Intestinal short chain fatty acids and their link with diet and human health. Front. Microbiol. 7:185. doi: 10.3389/fmicb.2016.00185, PMID: 26925050 PMC4756104

[ref14] Dong H. Liu B. Li A. Iqbal M. Wu Q. (2021). Microbiome analysis reveals the attenuation effect of lactobacillus from yaks on diarrhea via modulation of gut microbiota. Front. Cell. Infect. Microbiol. 10:610781. doi: 10.3389/fcimb.2020.610781, PMID: 33665171 PMC7920975

[ref15] Doron I. Mesko M. Li X. V. Kusakabe T. Leonardi I. Shaw D. G.. (2021). Mycobiota-induced IgA antibodies regulate fungal commensalism in the gut and are dysregulated in Crohn's disease. Nat. Microbiol. 6, 1493–1504. doi: 10.1038/s41564-021-00983-z, PMID: 34811531 PMC8622360

[ref16] Downes J. Dewhirst F. E. Tanner A. C. R. Wade W. G. (2013). Description of *Alloprevotella rava* gen. Nov., sp. nov., isolated from the human oral cavity, and reclassification of prevotella tannerae Moore et al. 1994 as *Alloprevotella tannerae* gen. Nov., comb. nov. Int. J. Syst. Evol. Microbiol. 63, 1214–1218. doi: 10.1099/ijs.0.041376-0, PMID: 22753527 PMC3709537

[ref17] Edgar R. C. (2013). UPARSE: highly accurate OTU sequences from microbial amplicon reads. Nat. Methods 10, 996–998. doi: 10.1038/nmeth.2604, PMID: 23955772

[ref18] Fata G. L. Weber P. Mohajeri M. H. (2018). Probiotics and the gut Iimmune system: indirect regulation. Probiotics and Antimicrobial Proteins 10, 11–21. doi: 10.1007/s12602-017-9322-6, PMID: 28861741 PMC5801397

[ref19] Feng Y. H. Wang Y. Wang P. Huang Y. L. Wang F. J. (2018). Short-chain fatty acids manifest stimulative and protective effects on intestinal barrier function through the inhibition of NLRP3 inflammasome and autophagy. Cell. Physiol. Biochem. 49, 190–205. doi: 10.1159/000492853, PMID: 30138914

[ref20] Fiers W. D. Leonardi I. Iliev I. D. (2020). From birth and throughout life: fungal microbiota in nutrition and metabolic health. Annu. Rev. Nutr. 40, 323–343. doi: 10.1146/annurev-nutr-013120-043659, PMID: 32680437 PMC7529963

[ref21] Gu J. L. Wang Y. Z. Liu S. Y. Yu G. J. Zhang T. Lu H. (2016). Gut microbiota community adaption during young children fecal microbiota transplantation by 16s rDNA sequencing. Neuruting 206, 66–72. doi: 10.1016/j.neucom.2016.01.095

[ref22] Guo H. H. Xue S. H. Nasir M. Lv J. L. Gu J. (2018). Role of bentonite on the mobility of antibiotic resistance genes, and microbial community in oxytetracycline and cadmium contaminated soil. Front. Microbiol. 9:2722. doi: 10.3389/fmicb.2018.02722, PMID: 30546348 PMC6279858

[ref23] Hamilton M. J. Weingarden A. R. Sadowsky M. J. Khoruts A. (2012). Standardized frozen preparation for transplantation of fecal microbiota for recurrent clostridium difficile infection. Am. J. Gastroenterol. 107, 761–767. doi: 10.1038/ajg.2011.482, PMID: 22290405

[ref24] He Z. Cui B. T. Zhang T. Li P. Long C. Y. Ji G. Z.. (2017). Fecal microbiota transplantation cured epilepsy in a case with Crohn's disease: the first report. World J. Gastroenterol. 23, 3565–3568. doi: 10.3748/wjg.v23.i19.3565, PMID: 28596693 PMC5442093

[ref25] Hu J. Chen L. L. Tang Y. M. Xie C. L. Xu B. Y. Shi M.. (2018a). Standardized preparation for fecal microbiota transplantation in pigs. Front. Microbiol. 9:1328. doi: 10.3389/fmicb.2018.01328, PMID: 29971061 PMC6018536

[ref26] Hu J. Ma L. B. Nie Y. F. Chen J. W. Zheng W. Y. Wang X. K.. (2018b). A microbiota-derived bacteriocin targets the host to confer diarrhea resistance in early-weaned piglets. Cell Host Microbe 24, 817–832.e8. doi: 10.1016/j.chom.2018.11.006, PMID: 30543777

[ref27] Jang K. B. Kim S. W. (2019). Supplemental effects of dietary nucleotides on intestinal health and growth performance of newly weaned pigs. J. Anim. Sci. 97, 4875–4882. doi: 10.1093/jas/skz334, PMID: 31665463 PMC6915224

[ref28] Kaźmierczak-Siedlecka K. Roviello G. Catalano M. Polom K. (2021). Gut microbiota modulation in the context of immune-related aspects of *Lactobacillus* spp. and *Bifidobacterium* spp. in gastrointestinal cancers. Nutrients 13:2674. doi: 10.3390/nu13082674, PMID: 34444834 PMC8401094

[ref29] Kim C. H. Park J. Kim M. (2014). Gut microbiota-derived short-chain fatty acids, T cells, and inflammation. Immune. Netw. 14, 277–288. doi: 10.4110/in.2014.14.6.277, PMID: 25550694 PMC4275385

[ref30] Kim H. S. Whon T. W. Sung H. Jeong Y. S. Jung E. S. Shin N. R.. (2021). Longitudinal evaluation of fecal microbiota transplantation for ameliorating calf diarrhea and improving growth performance. Nat. Commun. 12:161. doi: 10.1038/s41467-020-20389-5, PMID: 33420064 PMC7794225

[ref31] Klein-Jöbstl D. Schornsteiner E. Mann E. Wagner M. Drillich M. Schmitz-Esser S. (2014). Pyrosequencing reveals diverse fecal microbiota in Simmental calves during early development. Front. Microbiol. 5:622. doi: 10.3389/fmicb.2014.00622, PMID: 25452753 PMC4233928

[ref32] Leshem A. Horesh N. Elinav E. (2019). Fecal microbial transplantation and its potential application in cardiometabolic syndrome. Front. Immunol. 10:1341. doi: 10.3389/fimmu.2019.01341, PMID: 31258528 PMC6587678

[ref33] Li Y. Guo Y. Wen Z. Jiang X. Ma X. (2018). Weaning stress perturbs gut microbiome and its metabolic profile in piglets. Sci. Rep. 8:18068. doi: 10.1038/s41598-018-33649-8, PMID: 30584255 PMC6305375

[ref34] Li Y. Shi M. Zhang T. Hu X. Liu S. (2020). Dynamic changes in intestinal microbiota in young forest musk deer during weaning. Peer J. 8:e8923. doi: 10.7717/peerj.8923, PMID: 32322440 PMC7161571

[ref35] Li S. S. Zhu A. Benes V. Costea P. I. Hercog R. Hildebrand F.. (2016). Durable coexistence of donor and recipient strains after fecal microbiota transplantation. Science 352, 586–589. doi: 10.1126/science.aad8852, PMID: 27126044

[ref36] Listed N. (2014). Antibiotic resistance-the need for global solutions. Br. Dent. J. 216, 1057–1098. doi: 10.1038/sj.bdj.2014.77

[ref37] Liu Y. C. Cheng X. Zhen W. R. Zeng D. Qu L. J. Wang Z.. (2021b). Yeast culture improves egg quality and reproductive performance of aged breeder layers by regulating gut microbes. Front. Microbiol. 12:633276. doi: 10.3389/fmicb.2021.633276, PMID: 33815314 PMC8018237

[ref38] Liu J. Y. Ding L. Zhai X. Wang D. M. Xiao C. Hui X. Y.. (2022). Maternal dietary betaine prevents high-fat diet-induced metabolic disorders and gut microbiota alterations in mouse dams and offspring from young to adult. Front. Microbiol. 13:809642. doi: 10.3389/fmicb.2022.809642, PMID: 35479641 PMC9037091

[ref39] Liu X. Mao B. Gu J. Wu J. Chen W. (2021a). Blautia – a new functional genus with potential probiotic properties? Gut Microbes 13, 1–21. doi: 10.1080/19490976.2021.1875796, PMID: 33525961 PMC7872077

[ref40] Liu C. Zhang L. Fu H. Li W. Zhang Y. (2019). Relationship research between fecal microbes and short chain fatty acid between wild yak and domestic yak. Acta Theriol. Sin. 39, 1–7. doi: 10.16829/j.slxb.150219

[ref41] Luo Y. H. Li J. Y. Zhou H. Yu B. He J. Wu A.. (2021). The highlighted nutritional significance of intestinal fungi: alteration of dietary carbohydrate composition triggers the shifts of colonic fungal community in a pig model. Appl. Environ. Microbiol. 87:e00038-21. doi: 10.1128/AEM.00038-21, PMID: 33712429 PMC8117771

[ref42] Mago T. Salzberg S. L. (2011). Flash: fast length adjustment of short reads to improve genome assemblies. Bioinformatics 27, 2957–2963. doi: 10.1093/bioinformatics/btr507, PMID: 21903629 PMC3198573

[ref43] Malmuthuge N. Liang G. Guan L. L. (2019). Regulation of rumen development in neonatal ruminants through microbial metagenomes and host transcriptomes. Genome Biol. 20:172. doi: 10.1186/s13059-019-1786-0, PMID: 31443695 PMC6708143

[ref44] Matthias H. Alexander S. Rob E. Tae-Wan K. Harshal C. Gary S.. (2011). Metagenomic discovery of biomass-degrading genes and genomes from cow rumen. Science 331, 463–467. doi: 10.1126/science.1200387, PMID: 21273488

[ref45] Mcguirk S. M. (2008). Disease management of dairy calves and heifers. Vet. Clin. North Am. Food Anim. Pract. 24, 139–153. doi: 10.1016/j.cvfa.2007.10.003, PMID: 18299036 PMC7135781

[ref46] McKenney P. T. Pamer E. G. (2015). From hype to hope: the gut microbiota in enteric infectious disease. Cells 163, 1326–1332. doi: 10.1016/j.cell.2015.11.032, PMID: 26638069 PMC4672394

[ref47] Muscato T. V. Tedeschi L. O. Russell J. B. (2002). The effect of ruminal fluid preparations on the growth and health of newborn, milk-fed dairy calves. J. Dairy Sci. 85, 648–656. doi: 10.3168/jds.S0022-0302(02)74119-2, PMID: 11949870

[ref48] Pempek J. A. Watkins L. R. Bruner C. E. Habing G. (2019). A multisite, randomized field trial to evaluate the influence of lactoferrin on the morbidity and mortality of dairy calves with diarrhea. J. Dairy Sci. 102, 9259–9267. doi: 10.3168/jds.2019-16476, PMID: 31400894 PMC7094274

[ref49] Qin R. B. Wang J. Chao C. Yu J. L. Copeland L. Wang S. J.. (2021). RS5 produced more butyric acid through regulating the microbial community of human gut microbiota. J. Agric. Food Chem. 69, 3209–3218. doi: 10.1021/acs.jafc.0c08187, PMID: 33630575

[ref50] Samantha Y. Nicole R. Suchita P. Hayley D. Mulder I. E. Nie D. (2018). Human gut bacteria as potent class I histone deacetylase inhibitors *in vitro* through production of butyric acid and valeric acid. PLoS One 13:e0201073. doi: 10.1371/journal.pone.0201073, PMID: 30052654 PMC6063406

[ref51] Sanders M. E. Merenstein D. J. Reid G. Gibson G. R. Rastall R. A. (2019). Probiotics and prebiotics in intestinal health and disease: from biology to the clinic. Nat. Rev. Gastroenterol. Hepatol. 16, 605–616. doi: 10.1038/s41575-019-0173-3, PMID: 31296969

[ref52] Serino M. (2019). SCFAs – the thin microbial metabolic line between good and bad. Nat. Rev. Endocrinol. 15, 318–319. doi: 10.1038/s41574-019-0205-7, PMID: 30976118

[ref53] Shao Y. Wang Y. Yuan Y. Xie Y. (2021). A systematic review on antibiotics misuse in livestock and aquaculture and regulation implications in China. Sci. Total Environ. 798:149205. doi: 10.1016/j.scitotenv.2021.149205, PMID: 34375247

[ref54] Smillie C. S. Sauk J. Gevers D. Friedman J. Sung J. Youngster I.. (2018). Straintracking reveals the determinants of bacterial engraftment in the human gut following fecal microbiota transplantation. Cell Host Microbe 23, 229–240.e5. doi: 10.1016/j.chom.2018.01.003, PMID: 29447696 PMC8318347

[ref55] Smits L. P. Bouter K. Vos W. D. Borody T. J. Nieuwdorp M. (2013). Therapeutic potential of fecal microbiota transplantation. Gastroenterology 145, 946–953. doi: 10.1053/j.gastro.2013.08.058 24018052

[ref01] Stackebrandt E. Goebel B. M. (1994). Taxonomic note: a place for DNA-DNA reassociation and 16S rRNA sequence analysis in the present species definition in bacteriology. Int. J. Syst. Bacteriol. 44, 846–849. doi: 10.1099/00207713-44-4-846

[ref56] Su Y. Li X. L. Li D. Y. Sun J. (2021). Fecal microbiota transplantation shows marked shifts in the multi-omic profiles of porcine post-weaning diarrhea. Front. Microbiol. 12:619460. doi: 10.3389/fmicb.2021.619460, PMID: 33708182 PMC7940351

[ref57] Tan J. Li Z. Zhou B. Dong M. Xia H. Han L.. (2018). Extracellular enzymatic activities of yeasts isolated from Fuxian Lake and Xingyun Lake in Yunnan plateau. Microbiol. China 45, 302–313. doi: 10.13344/j.microbiol.china.170262

[ref58] Tang Y. M. (2019). *Effect of Fecal Microbiota Transplantation of Meishan pig on Follicle Development in Landrace×Yorkshire Gilts. [Doctoral’s Thesis], Huazhong Agricultural University*.

[ref59] Uyeno Y. Sekiguchi Y. Kamagata Y. (2010). rRNA-based analysis to monitor succession of faecal bacterial communities in Holstein calves. Lett. Appl. Microbiol. 51, 570–577. doi: 10.1111/j.1472-765X.2010.02937.x, PMID: 20849397

[ref60] Verbeke K. A. Boobis A. R. Chiodini A. Edwards C. A. Franck A. Kleerebezem M.. (2015). Towards microbial fermentation metabolites as markers for health benefits of prebiotics. Nutr. Res. Rev. 28, 42–66. doi: 10.1017/S0954422415000037, PMID: 26156216 PMC4501371

[ref61] Wang D. Du Y. Huang S. You Z. Zheng D. Liu Y. (2021). Combined supplementation of sodium humate and glutamine reduced diarrhea incidence of weaned calves by intestinal microbiota and metabolites changes. J. Anim. Sci. 99:skab305. doi: 10.1093/jas/skab305, PMID: 34673954 PMC8599267

[ref62] Wang Y. W. Song J. L. Zhai Y. Zhang C. Gerritsen J. Wang H. M.. (2015). Romboutsia sedimentorum sp nov., isolated from an alkaline-saline lake sediment and emended description of the genus Romboutsia. Int. J. Syst. Evol. Microbiol. 65, 1193–1198. doi: 10.1099/ijs.0.000079, PMID: 25609678

[ref63] Wei Z. Yang T. Friman V. P. Xu Y. Shen Q. Jousset A. (2015). Trophic network architecture of root-associated bacterial communities determines pathogen invasion and plant health. Nat. Commun. 6:8413. doi: 10.1038/ncomms9413, PMID: 26400552 PMC4598729

[ref64] William M. L. Rodrigo M. Raaijmakers J. M. Mui T. S. (2018). Breeding for soil-borne pathogen resistance impacts active rhizosphere microbiome of common bean. ISME J. 12, 3038–3042. doi: 10.1038/s41396-018-0234-6, PMID: 30018368 PMC6246553

[ref65] Wu Z. H. (2018). *Effects of Fecal Microbiota Transplantation on Intestinal Barrier Function and Microbiota Establishment in Calves with Failure of Passive Immune Transfer. [Doctoral’s Thesis], China Agricultural University.*

[ref66] Wu Y. He F. Zhang C. Zhang Q. Lin J. (2021). Melatonin alleviates titanium nanoparticles induced osteolysis via activation of butyrate/GPR109A signaling pathway. J. Nanobiotechnol. 19:170. doi: 10.1186/s12951-021-00915-3, PMID: 34092246 PMC8182936

[ref67] Xiang Q. Wu X. Pan Y. Wang L. Wei H. (2020). Early intervention using fecal microbiota transplantation combined with probioticsinfluence the growth performance, Ddiarrhea, and intestinal barrier function of piglets. Appl. Sci. 10:568. doi: 10.3390/app10020568

[ref68] Zhang C. Y. Deng Y. C. Zheng J. F. Zhang Y. Yang L. H. Liao C. J.. (2019). The application of the QuEChERS methodology in the determination of antibiotics in food: a review. TrAC Trends Anal. Chem. 118, 517–537. doi: 10.1016/j.trac.2019.06.012

[ref69] Zhang J. B. Wang P. Dingkao R. Q. Du M. Ahmad A. A. Liang Z. Y.. (2021). Fecal microbiota dynamics reveal the feasibility of early weaning of yak calves under conventional grazing system. Biology 11:31. doi: 10.3390/biology11010031, PMID: 35053029 PMC8773362

[ref70] Zhou S. S. Luo R. B. Gong G. Wang Y. F. Gesang Z. M. Wang K.. (2020). Characterization of metagenome-assembled genomes and carbohydrate-degrading genes in the gut microbiota of Tibetan pig. Front. Microbiol. 11:595066. doi: 10.3389/fmicb.2020.595066, PMID: 33424798 PMC7785962

[ref71] Zhu J. T. Lin H. Wu X. Li Z. W. Lin A. Y. (2019). Metataxonomics of internal transcribed spacer amplicons in cerebrospinal fluid for diagnosing and genotyping of cryptococcal meningitis. Chin. Med. J. 132, 2827–2834. doi: 10.1097/CM9.0000000000000541, PMID: 31856054 PMC6940084

