# Supplementary material for: Effects of fecal microbiota transplantation from yaks on weaning diarrhea, fecal microbiota composition, microbial network structure and functional pathways in Chinese Holstein calves
Source: Front Microbiol. 2022 Sep 23;13:898505. doi: 10.3389/fmicb.2022.898505 (PMC9537452; doi:10.3389/fmicb.2022.898505)
Supplement: Supplementary file 1 [file Data_Sheet_1.doc]

Supplementary Table 1. Relative abundance (%) of fecal microbiota composition in fecal microbial transplant donors

| **Bacteria** | | | | | | | |
| --- | --- | --- | --- | --- | --- | --- | --- |
| **Phylum** | | | | | | | |
| Firmicutes | 76.33 | Bacteroidetes | 22.44 | Actinobacteria | 0.37 | TM7 | 0.33 |
| Proteobacteria | 0.18 | Verrucomicrobia | 0.10 | Synergistetes | 0.05 | Chloroflexi | 0.04 |
| Tenericutes | 0.03 | Others | 0.12 |  |  |  |  |
| **Genus** |  |  |  |  |  |  |  |
| 5-7N15 | 9.60 | Dorea | 3.91 | Oscillospira | 3.86 | Roseburia | 2.77 |
| [Clostridium] | 1.61 | SMB53 | 0.96 | Clostridiaceae_Clostridium | 0.94 | CF231 | 0.81 |
| Coprococcus | 0.70 | Coprobacillus | 0.56 | Phascolarctobacterium | 0.54 | Ruminococcaceae_Ruminococcus | 0.47 |
| Mogibacterium | 0.45 | [Eubacterium] | 0.40 | [Prevotella] | 0.28 | Blautia | 0.26 |
| Dehalobacterium | 0.23 | Prevotella | 0.17 | Solibacillus | 0.17 | Paludibacter | 0.16 |
| Others | 71.16 |  |  |  |  |  |  |
| **Fungi** |  |  |  |  |  |  |  |
| **Phylum** |  |  |  |  |  |  |  |
| Ascomycota | 85.50 | Basidiomycota | 3.21 | Mortierellomycota | 0.44 | Neocallimastigomycota | 0.11 |
| Rozellomycota | 0.08 | Mucoromycota | 0.06 | Olpidiomycota | 0.03 | Others | 10.58 |
| **Genus** |  |  |  |  |  |  |  |
| Preussia | 21.75 | Neoascochyta | 0.83 | Mortierella | 0.44 | Coprinopsis | 0.32 |
| Sporormiella | 3.40 | Aspergillus | 0.80 | Pichia | 0.43 | Urocystis | 0.31 |
| Pseudogymnoascus | 3.39 | Naganishia | 0.63 | Ustilago | 0.42 | Fusarium | 0.28 |
| Podospora | 1.99 | Lycoperdon | 0.62 | Plenodomus | 0.39 | Venturia | 0.26 |
| Humicola | 1.86 | Thelebolus | 0.52 | Sarocladium | 0.33 | Microbotryum | 0.23 |
| Others | 60.80 |  |  |  |  |  |  |

Supplementary Table 2. FMT recipient cattle selection criteria

| 1. Male and female calves aged 47-53 days 2. The weight, appearance and growth performance of cattle are similar 3. No recessive disease gene was found by pedigree relationship 4. No history of taking antibacterial agents 5. There are no genetic defects and diseases. 6. No common bovine infectious diseases such as foot-and-mouth disease, mad cow disease, brucellosis, tuberculosis, etc. were found |
| --- |

Supplementary Table 3.Ingredients of the starter concentrate (%)

| Ingredient | Content |
| --- | --- |
| Corn | 55.200 |
| Soybean meal | 18.500 |
| Corn gluten meal | 10.000 |
| DGGS | 13.000 |
| Limestone | 1.800 |
| NaCl | 0.500 |
| Premix^1^ | 1.000 |
| Total | 100.000 |
| ^1^Premix provides the following per kg of the starter diet: VA 15,000 IU, VD 5000 IU, VE 50.000 mg, Fe 90.000 mg, Cu 12.500 mg, Mn 30.000 mg, Zn 90.000 mg, Se 0.300 mg, I 1.000 mg, Co 0.500 mg | |

Supplementary Table 4. Relative abundance (%) of major fecal bacterial compositions at the phylum level in Holstein cows treated with different concentrations of FMT at four time points after weaning

Experimental Treatments: NC=no supplementation; Control=normal saline; LFMT=1×10^8^ CFU/mL bacterial solution/calf/time; HFMT=1×10^9^ CFU/mL bacterial solution/calf/time ; SFMT=sterilized bacterial solution ;

| **Phylum** | | **Experimental Treatments (Trt)** | | | | | **SEM** | **P-value** | | | | | | |
| --- | --- | --- | --- | --- | --- | --- | --- | --- | --- | --- | --- | --- | --- | --- |
|  |  | **NC** | **Control** | **LFMT** | **HFMT** | **SFMT** |  | **Trt** | **Time** | **Trt×Time** | **NC×Control** | **Control×**  **LFMT** | **Control×**  **HFMT** | **Control×**  **SFMT** |
| Firmicutes | | | | | | | | | | | | | | |
| 5d | 50.75 | | 50.98 | 54.41 | 64.33 | 53.73 | 3.15 | 0.01 | 0.33 | 0.08 | 0.99 | 0.75 | 0.31 | 0.80 |
| 10d | 54.02^a^ | | 39.46^b^ | 56.35^ab^ | 57.10^a^ | 54.99^a^ | 2.90 |  |  |  | 0.75 | 0.31 | 0.80 | 0.01 |
| 15d | 52.15^ab^ | | 51.95^b^ | 55.62^a^ | 51.18^ab^ | 54.16^ab^ | 2.92 |  |  |  | 0.10 | 0.05 | 0.38 | 0.05 |
| 20d | 27.51^c^ | | 49.62^ab^ | 48.35^b^ | 53.23^ab^ | 60.360^a^ | 2.80 |  |  |  | 0.01 | 0.86 | 0.53 | 0.13 |
| Bacteroidota | | | | | | | | | | | | | | |
| 5d | 41.92 | | 41.91 | 41.32 | 27.66 | 38.79 | 3.60 | 0.11 | 0.89 | 0.32 | 1.00 | 0.96 | 0.12 | 0.75 |
| 10d | 43.27 | | 49.70 | 37.12 | 33.30 | 42.21 | 2.83 |  |  |  | 0.37 | 0.33 | 0.11 | 0.20 |
| 15d | 39.92^b^ | | 55.24^a^ | 22.91^b^ | 44.74^ab^ | 38.24^b^ | 2.99 |  |  |  | 0.01 | 0.01 | 0.14 | 0.02 |
| 20d | 45.58 | | 35.30 | 32.93 | 43.59 | 34.97 | 2.90 |  |  |  | 0.34 | 0.82 | 0.40 | 0.96 |
| Actinobacteriota | | | | | | | | | | | | | | |
| 5d | 4.53 | | 4.97 | 2.14 | 4.18 | 5.76 | 0.84 | 0.35 | 0.16 | 0.54 | 0.90 | 0.21 | 0.83 | 0.79 |
| 10d | 0.60^c^ | | 7.19^a^ | 2.04^bc^ | 4.16^bc^ | 1.32^b^ | 0.71 |  |  |  | 0.00 | 0.00 | 0.01 | 0.00 |
| 15d | 1.90 | | 2.94 | 1.61 | 1.11 | 5.63 | 0.73 |  |  |  | 0.48 | 0.25 | 0.12 | 0.44 |
| 20d | 5.23 | | 2.11 | 10.00 | 1.06 | 2.92 | 1.39 |  |  |  | 0.58 | 0.90 | 0.35 | 0.38 |
| Spirochaetota | | | | | | | | | | | | | | |
| 5d | 1.89 | | 0.89 | 0.75 | 0.76 | 0.04 | 0.36 | 0.01 | 0.08 | 0.01 | 0.58 | 0.84 | 0.94 | 0.17 |
| 10d | 0.91^ab^ | | 2.76^ab^ | 4.06^a^ | 0.35^ab^ | 0.19^b^ | 0.48 |  |  |  | 0.16 | 0.53 | 0.12 | 0.06 |
| 15d | 4.63 | | 0.07 | 9.56 | 5.08 | 0.20 | 1.42 |  |  |  | 0.13 | 0.11 | 0.09 | 0.48 |
| 20d | 17.95^ab^ | | 0.01^b^ | 3.43^a^ | 1.41^ab^ | 0.28^b^ | 2.18 |  |  |  | 0.09 | 0.01 | 0.17 | 0.12 |
| Proteobacteria | | | | | | | | | | | | | | |
| 5d | 0.41 | | 1.08 | 0.39 | 0.83 | 1.28 | 0.22 | 1.00 | 0.09 | 0.17 | 0.49 | 0.48 | 0.96 | 0.85 |
| 10d | 0.60^ab^ | | 0.14^c^ | 0.28^bc^ | 3.13 ^abc^ | 1.19 ^a^ | 0.54 |  |  |  | 0.02 | 0.26 | 0.09 | 0.01 |
| 15d | 0.37^b^ | | 0.76^ab^ | 0.68^ab^ | 0.68^ab^ | 1.53^a^ | 0.17 |  |  |  | 0.40 | 0.88 | 0.91 | 0.23 |
| 20d | 2.72^ab^ | | 2.95^ab^ | 3.65^a^ | 0.32^b^ | 1.01^b^ | 0.58 |  |  |  | 0.93 | 0.72 | 0.22 | 0.31 |
| Cyanobacteria | | | | | | | | | | | | | | |
| 5d | 0.15^b^ | | 0.08 ^ab^ | 0.10^ab^ | 1.76^ab^ | 0.29^b^ | 0.23 | 0.06 | 0.02 | 0.01 | 0.16 | 0.70 | 0.08 | 0.04 |
| 10d | 0.06^a^ | | 0.54ab | 0.06^ab^ | 0.20 ^ab^ | 0.01^b^ | 0.07 |  |  |  | 0.14 | 0.14 | 0.38 | 0.10 |
| 15d | 0.03 | | 0.03 | 0.04 | 0.05 | 0.02 | 0.01 |  |  |  | 0.78 | 0.61 | 0.78 | 0.38 |
| 20d | 0.01^c^ | | 0.11^abc^ | 0.13^abc^ | 0.04^b^ | 0.13^a^ | 0.02 |  |  |  | 0.27 | 0.86 | 0.49 | 0.81 |

^a,b,c^ In the same row, values with different letter superscripts mean significant difference (*P*<0.05)

Supplementary Table 5. Relative abundances (%) of major fecal bacterial compositions at the genus level in Holstein cows treated with different concentrations of FMT at four time points after weaning

| **Genus** | | **Experimental Treatments (Trt)** | | | | | **SEM** | **P-value** | | | | | | |
| --- | --- | --- | --- | --- | --- | --- | --- | --- | --- | --- | --- | --- | --- | --- |
|  |  | **NC** | **Control** | **LFMT** | **HFMT** | **SFMT** |  | **Trt** | **Time** | **Trt×Time** | **NC×Control** | **Control×**  **LFMT** | **Control×**  **HFMT** | **Control×**  **SFMT** |
| norank_f__Muribaculaceae | | | | | | | | | | | | | | |
| 5d | 22.03^b^ | | 7.39^b^ | 16.70^a^ | 6.40^ab^ | 5.90^b^ | 2.43 | 0.50 | 0.18 | 0.02 | 0.18 | 0.05 | 0.23 | 0.26 |
| 10d | 8.79^a^ | | 9.79^ab^ | 6.04^b^ | 7.37^ab^ | 6.81^ab^ | 0.70 |  |  |  | 0.72 | 0.18 | 0.53 | 0.38 |
| 15d | 8.42^ab^ | | 14.13^a^ | 4.31^b^ | 16.26^a^ | 13.99^ab^ | 1.95 |  |  |  | 0.25 | 0.05 | 0.51 | 0.99 |
| 20d | 10.81^ab^ | | 3.22^b^ | 6.96^ab^ | 8.21^a^ | 12.32^a^ | 1.10 |  |  |  | 0.06 | 0.08 | 0.00 | 0.01 |
| UCG-005 | | | | | | | | | | | | | | |
| 5d | 12.58 | | 9.27 | 8.01 | 10.02 | 7.15 | 1.34 | ＜0.01 | 0.09 | ＜0.01 | 0.40 | 0.78 | 0.82 | 0.58 |
| 10d | 10.37^a^ | | 3.96^b^ | 4.08^b^ | 10.84^a^ | 7.26^ab^ | 0.94 |  |  |  | 0.01 | 0.95 | 0.00 | 0.31 |
| 15d | 17.57 | | 8.59 | 7.14 | 13.68 | 9.68 | 1.49 |  |  |  | 0.09 | 0.69 | 0.07 | 0.80 |
| 20d | 1.93^c^ | | 5.18^bc^ | 5.83^b^ | 23.33^a^ | 6.66^b^ | 1.70 |  |  |  | 0.12 | 0.74 | 0.00 | 0.53 |
| Rikenellaceae_RC9_gut_group | | | | | | | | | | | | | | |
| 5d | 6.16^ac^ | | 2.72^b^ | 11.72^a^ | 2.09^c^ | 9.30^a^ | 0.96 | 0.86 | 0.02 | 0.03 | 0.05 | 0.00 | 0.02 | 0.00 |
| 10d | 10.27 | | 9.19 | 11.02 | 7.18 | 12.26 | 1.47 |  |  |  | 0.84 | 0.70 | 0.68 | 0.50 |
| 15d | 9.03^a^ | | 14.19^ab^ | 8.53^ab^ | 18.59^ab^ | 1.83^b^ | 2.29 |  |  |  | 0.38 | 0.46 | 0.98 | 0.05 |
| 20d | 1.99^c^ | | 5.81^abc^ | 4.42^b^ | 7.19^a^ | 5.57^abc^ | 0.67 |  |  |  | 0.08 | 0.49 | 0.79 | 0.93 |
| Bacteroides | | | | | | | | | | | | | | |
| 5d | 5.75 | | 7.44 | 3.88 | 8.26 | 4.93 | 1.10 | 0.54 | 0.28 | 0.80 | 0.68 | 0.26 | 0.54 | 0.37 |
| 10d | 8.75 | | 12.04 | 3.91 | 8.37 | 10.62 | 1.54 |  |  |  | 0.57 | 0.18 | 0.82 | 0.84 |
| 15d | 9.37^a^ | | 4.01^b^ | 3.52^b^ | 4.05^ab^ | 8.90^ab^ | 0.92 |  |  |  | 0.05 | 0.72 | 0.89 | 0.15 |
| 20d | 6.70 | | 8.79 | 9.80 | 7.05 | 10.21 | 1.48 |  |  |  | 0.58 | 0.85 | 0.87 | 0.82 |
| Blautia | | | | | | | | | | | | | | |
| 5d | 3.43^ab^ | | 2.44^b^ | 5.92^a^ | 19.04^ab^ | 5.70^a^ | 2.32 | 0.12 | 0.24 | 0.06 | 0.39 | 0.05 | 0.09 | 0.01 |
| 10d | 4.08 | | 2.79 | 6.76 | 7.89 | 3.30 | 1.11 |  |  |  | 0.15 | 0.29 | 0.46 | 0.49 |
| 15d | 2.08^b^ | | 2.61^b^ | 9.16^ab^ | 2.11^b^ | 6.72^a^ | 1.04 |  |  |  | 0.55 | 0.19 | 0.59 | 0.01 |
| 20d | 3.24^ab^ | | 4.38^a^ | 4.95^a^ | 1.22^b^ | 4.19^a^ | 0.40 |  |  |  | 0.41 | 0.59 | 0.01 | 0.83 |
| unclassified_f__Lachnospiraceae | | | | | | | | | | | | | | |
| 5d | 3.36 | | 4.31 | 2.34 | 3.44 | 1.60 | 0.43 | 0.77 | 0.04 | 0.26 | 0.56 | 0.16 | 0.69 | 0.05 |
| 10d | 5.71 | | 3.54 | 3.96 | 2.99 | 4.20 | 0.54 |  |  |  | 0.16 | 0.83 | 0.98 | 0.66 |
| 15d | 7.57 | | 4.04 | 7.54 | 4.34 | 3.35 | 0.92 |  |  |  | 0.30 | 0.26 | 0.81 | 0.48 |
| 20d | 1.95^b^ | | 3.40^ab^ | 2.76^ab^ | 4.85^a^ | 4.50^a^ | 0.37 |  |  |  | 0.09 | 0.44 | 0.31 | 0.40 |
| norank_f__norank_o__Clostridia_UCG-014 | | | | | | | | | | | | | | |
| 5d | 2.56 | | 1.84 | 2.00 | 2.91 | 2.55 | 0.42 | ＜0.01 | ＜0.01 | 0.01 | 0.64 | 0.86 | 0.40 | 0.40 |
| 10d | 5.29^ab^ | | 7.67^a^ | 2.68^bc^ | 2.04^c^ | 7.77^a^ | 0.64 |  |  |  | 0.13 | 0.00 | 0.00 | 0.95 |
| 15d | 1.48^c^ | | 4.65^a^ | 2.49^bc^ | 3.34^ab^ | 3.39^abc^ | 0.39 |  |  |  | 0.01 | 0.05 | 0.22 | 0.44 |
| 20d | 1.37 | | 5.43 | 2.86 | 2.52 | 6.16 | 0.58 |  |  |  | 0.08 | 0.27 | 0.20 | 0.73 |
| Prevotella | | | | | | | | | | | | | | |
| 5d | 0.29^b^ | | 2.99^ab^ | 0.81^b^ | 0.56^b^ | 5.89^a^ | 0.61 | ＜0.01 | ＜0.01 | ＜0.01 | 0.14 | 0.23 | 0.21 | 0.25 |
| 10d | 4.85^b^ | | 10.55^a^ | 1.42^b^ | 0.54^b^ | 2.80^b^ | 0.88 |  |  |  | 0.04 | 0.00 | 0.00 | 0.00 |
| 15d | 3.79 | | 6.11 | 2.04 | 1.51 | 2.92 | 0.62 |  |  |  | 0.35 | 0.12 | 0.09 | 0.20 |
| 20d | 7.61^a^ | | 5.87^a^ | 4.57^ab^ | 1.46^b^ | 1.50^b^ | 0.71 |  |  |  | 0.39 | 0.56 | 0.00 | 0.00 |
| Alloprevotella | | | | | | | | | | | | | | |
| 5d | 1.64^b^ | | 1.98^b^ | 3.10^b^ | 2.37^b^ | 8.67^a^ | 0.75 | 0.13 | 0.22 | ＜0.01 | 0.79 | 0.60 | 0.78 | 0.00 |
| 10d | 4.10^a^ | | 0.73^b^ | 10.41^ab^ | 0.90^b^ | 4.39^ab^ | 1.32 |  |  |  | 0.02 | 0.12 | 0.54 | 0.12 |
| 15d | 0.51 | | 4.53 | 0.59 | 0.46 | 3.53 | 0.60 |  |  |  | 0.10 | 0.10 | 0.14 | 0.71 |
| 20d | 3.16^abc^ | | 6.70^a^ | 1.82^b^ | 2.92^abc^ | 0.91^c^ | 0.56 |  |  |  | 0.10 | 0.02 | 0.08 | 0.01 |
| Treponema | | | | | | | | | | | | | | |
| 5d | 1.86 | | 0.88 | 0.64 | 0.13 | 0.02 | 0.35 | 0.01 | 0.07 | 0.01 | 0.58 | 0.72 | 0.30 | 0.16 |
| 10d | 0.90^ab^ | | 2.55^ab^ | 3.47^a^ | 0.10^b^ | 0.15^b^ | 0.43 |  |  |  | 0.18 | 0.61 | 0.08 | 0.05 |
| 15d | 4.62 | | 0.03 | 9.55 | 5.03 | 0.00 | 1.42 |  |  |  | 0.13 | 0.11 | 0.09 | 0.22 |
| 20d | 17.89^ab^ | | 0.00^b^ | 3.37^a^ | 1.19^ab^ | 0.23^b^ | 2.18 |  |  |  | 0.09 | 0.01 | 0.22 | 0.15 |
| norank_f__Eubacterium_coprostanoligenes_group | | | | | | | | | | | | | | |
| 5d | 2.58 | | 1.02 | 1.72 | 1.14 | 2.75 | 0.37 | 0.02 | 0.31 | 0.45 | 0.09 | 0.02 | 0.47 | 0.31 |
| 10d | 2.95 | | 1.89 | 0.78 | 2.59 | 8.21 | 1.09 |  |  |  | 0.59 | 0.19 | 0.58 | 0.21 |
| 15d | 1.97 | | 2.01 | 1.21 | 3.09 | 1.77 | 0.34 |  |  |  | 0.97 | 0.28 | 0.86 | 0.77 |
| 20d | 2.08^ab^ | | 0.77^b^ | 1.49^ab^ | 1.35^ab^ | 4.44^a^ | 0.42 |  |  |  | 0.15 | 0.35 | 0.29 | 0.04 |
| Bifidobacterium | | | | | | | | | | | | | | |
| 5d | 3.90^ab^ | | 3.82^ab^ | 1.25^a^ | 0.02^b^ | 4.93^a^ | 0.80 | 0.04 | 0.26 | 0.67 | 0.98 | 0.27 | 0.16 | 0.72 |
| 10d | 0.07^bc^ | | 5.73^a^ | 0.49^bc^ | 0.01^c^ | 0.03^b^ | 0.48 |  |  |  | 0.00 | 0.00 | 0.00 | 0.00 |
| 15d | 1.68^ab^ | | 0.54^a^ | 0.21^ab^ | 0.02^ab^ | 0.01^b^ | 0.23 |  |  |  | 0.32 | 0.18 | 0.06 | 0.04 |
| 20d | 5.43 | | 9.82 | 0.25 | 0.69 | 0.02 | 2.01 |  |  |  | 0.68 | 0.36 | 0.42 | 0.34 |
| Ruminococcus | | | | | | | | | | | | | | |
| 5d | 0.65^c^ | | 1.21^bc^ | 2.06^bc^ | 2.70^b^ | 7.37^a^ | 0.55 | ＜0.01 | ＜0.01 | ＜0.01 | 0.11 | 0.23 | 0.06 | 0.00 |
| 10d | 1.79^ab^ | | 1.16^b^ | 0.80^b^ | 3.05^ab^ | 2.98^a^ | 0.35 |  |  |  | 0.47 | 0.48 | 0.09 | 0.01 |
| 15d | 0.78 | | 0.48 | 1.49 | 2.58 | 1.47 | 0.34 |  |  |  | 0.41 | 0.06 | 0.17 | 0.15 |
| 20d | 2.07 | | 1.94 | 0.67 | 0.87 | 1.09 | 0.30 |  |  |  | 0.92 | 0.20 | 0.25 | 0.38 |
| Prevotellaceae_NK3B31_group | | | | | | | | | | | | | | |
| 5d | 0.18 | | 9.50 | 0.07 | 0.43 | 0.11 | 1.32 | 0.01 | 0.15 | ＜0.01 | 0.16 | 0.15 | 0.22 | 0.15 |
| 10d | 0.25^b^ | | 0.89^a^ | 0.47^ab^ | 0.06^b^ | 0.15^b^ | 0.08 |  |  |  | 0.00 | 0.10 | 0.00 | 0.00 |
| 15d | 1.37^ab^ | | 5.76^a^ | 0.44^b^ | 0.06^c^ | 0.38^bc^ | 0.57 |  |  |  | 0.06 | 0.02 | 0.04 | 0.02 |
| 20d | 3.77 | | 0.15 | 0.38 | 8.79 | 0.38 | 0.95 |  |  |  | 0.08 | 0.25 | 0.05 | 0.36 |
| Christensenellaceae_R-7_group | | | | | | | | | | | | | | |
| 5d | 4.82^a^ | | 2.82^abc^ | 1.88^abc^ | 1.34b | 0.25^c^ | 0.48 | 0.01 | 0.02 | 0.01 | 0.30 | 0.54 | 0.25 | 0.09 |
| 10d | 2.87^a^ | | 1.40^b^ | 0.55^b^ | 1.79^ab^ | 0.56^b^ | 0.27 |  |  |  | 0.01 | 0.06 | 0.76 | 0.16 |
| 15d | 2.20 | | 0.73 | 1.07 | 0.62 | 1.21 | 0.28 |  |  |  | 0.21 | 0.35 | 0.53 | 0.59 |
| 20d | 0.47^b^ | | 0.42^b^ | 1.04^ab^ | 1.00^a^ | 2.24^ab^ | 0.23 |  |  |  | 0.82 | 0.28 | 0.02 | 0.07 |
| Parabacteroides | | | | | | | | | | | | | | |
| 5d | 0.45^c^ | | 0.85^bc^ | 1.29^b^ | 2.46^a^ | 2.29^a^ | 0.18 | ＜0.01 | 0.58 | ＜0.01 | 0.05 | 0.23 | 0.00 | 0.00 |
| 10d | 1.83 | | 1.66 | 0.78 | 1.13 | 1.18 | 0.20 |  |  |  | 0.80 | 0.08 | 0.68 | 0.54 |
| 15d | 1.69^a^ | | 0.69^b^ | 0.94^b^ | 0.85^b^ | 1.96^ab^ | 0.18 |  |  |  | 0.00 | 0.25 | 0.43 | 0.12 |
| 20d | 1.08^bc^ | | 0.87^bc^ | 0.66^c^ | 1.89^a^ | 1.52^ab^ | 0.13 |  |  |  | 0.51 | 0.37 | 0.00 | 0.07 |
| Monoglobusx. | | | | | | | | | | | | | | |
| 5d | 0.49 | | 0.50 | 0.35 | 1.92 | 0.42 | 0.27 | ＜0.01 | 0.45 | 0.19 | 0.97 | 0.59 | 0.57 | 0.79 |
| 10d | 0.54 | | 0.18 | 0.18 | 5.58 | 0.98 | 0.68 |  |  |  | 0.01 | 0.99 | 0.06 | 0.09 |
| 15d | 0.42^a^ | | 0.46^b^ | 1.23^b^ | 4.12^ab^ | 0.87^ab^ | 0.43 |  |  |  | 0.85 | 0.16 | 0.02 | 0.34 |
| 20d | 0.93 | | 0.87 | 0.36 | 2.10 | 3.08 | 0.30 |  |  |  | 0.90 | 0.33 | 0.10 | 0.07 |
| Lactobacillus | | | | | | | | | | | | | | |
| 5d | 0.13 | | 0.00 | 9.84 | 0.02 | 0.00 | 1.40 | ＜0.01 | 0.45 | 0.58 | 0.16 | 0.15 | 0.18 | 0.14 |
| 10d | 0.00 | | 0.00 | 6.97 | 0.00 | 0.00 | 1.00 |  |  |  | 1.00 | 0.16 | 0.56 | 0.35 |
| 15d | 0.00 | | 0.00 | 8.26 | 0.00 | 0.01 | 1.15 |  |  |  | 0.06 | 0.15 | 0.10 | 0.36 |
| 20d | 0.00 | | 0.00 | 0.04 | 0.00 | 0.00 | 0.01 |  |  |  | 0.14 | 0.38 | 0.39 | 0.14 |
| Romboutsia | | | | | | | | | | | | | | |
| 5d | 1.94^ab^ | | 4.78^ab^ | 3.97^b^ | 0.02^a^ | 0.00^a^ | 0.70 | ＜0.01 | 0.08 | 0.22 | 0.39 | 0.79 | 0.19 | 0.14 |
| 10d | 0.79^b^ | | 0.20^b^ | 6.92^ab^ | 0.01^a^ | 0.04^ab^ | 0.95 |  |  |  | 0.11 | 0.15 | 0.05 | 0.08 |
| 15d | 0.16^b^ | | 0.00^a^ | 3.75^ab^ | 0.00^a^ | 0.01^ab^ | 0.52 |  |  |  | 0.04 | 0.14 | 0.41 | 0.13 |
| 20d | 0.02 | | 0.01 | 0.64 | 0.00 | 0.01 | 0.13 |  |  |  | 0.31 | 0.34 | 0.42 | 0.58 |

Experimental Treatments: NC=no supplementation; Control=normal saline; LFMT=1×10^8^ CFU/mL bacterial solution/calf/time; HFMT=1×10^9^ CFU/mL bacterial solution/calf/time; SFMT=sterilized bacterial solution;

^a,b,c^ In the same row, values with different letter superscripts mean significant difference (*P* < 0.05)

Supplementary Table 6. The topological properties of the network are obtained from different treatment groups

| Topological properties | 16S | | | | | ITS | |
| --- | --- | --- | --- | --- | --- | --- | --- |
|  | NC | Control | LFMT | HFMT | SFMT | Control | LFMT |
| Node_Num^1^ | 35 | 35 | 33 | 32 | 34 | 34 | 34 |
| Edge_Num ^2^ | 88 | 98 | 130 | 112 | 108 | 128 | 139 |
| Average node connectivity^3^ | 5.029 | 5.600 | 7.879 | 7.000 | 6.353 | 7.529 | 8.176 |
| 1.Number of OTUs with at least one correlation ≥ 0.5 or ≤ -0.5 and that are statistically significant (*P*< 0.05).  2.Number of strong and significant correlations between nodes.  3.Node connectivity showing how many connections (on average) each node has to another unique node in the network. | | | | | | | |
